# Evaluation of *GeneXpert vanA/vanB* in the early diagnosis of vancomycin-resistant enterococci infection

**DOI:** 10.1371/journal.pntd.0009869

**Published:** 2021-11-08

**Authors:** Zhuo-Lei Li, Qi-Bing Luo, Shan-Shan Xiao, Ze-Hong Lin, Ye-Ling Liu, Meng-Yi Han, Jing-Hua Zhong, Tian-Xing Ji, Xu-Guang Guo

**Affiliations:** 1 Department of Clinical Laboratory Medicine, The Third Affiliated Hospital of Guangzhou Medical University, Guangzhou, China; 2 Department of Clinical Medicine, The Second Clinical School of Guangzhou Medical University, Guangzhou, China; 3 Department of Pharmacy, Guangzhou Medical University School of Pharmaceutical Sciences, Guangzhou, China; 4 Department of Clinical Medicine, The Third Clinical School of Guangzhou Medical University, Guangzhou, China; 5 Department of Clinical Laboratory Medicine, The Second Affiliated Hospital of Guangzhou Medical University, Guangzhou, China; 6 Department of Key Laboratory for Major Obstetric Diseases of Guangdong Province, The Third Clinical School of Guangzhou Medical University, Guangzhou, China; 7 Department of Key Laboratory of Reproduction and Genetics of Guangdong Higher Education Institutes, The Third Clinical School of Guangzhou Medical University, Guangzhou, China; Creighton University, UNITED STATES

## Abstract

**Purpose:**

*Vancomycin-resistant enterococci* infection is a worrying worldwide clinical problem. To evaluate the accuracy of *GeneXpert vanA/vanB* in the diagnosis of VRE, we conducted a systematic review in the study.

**Methods:**

Experimental data were extracted from publications until May 03 2021 related to the diagnostic accuracy of *GeneXpert vanA/vanB* for *VRE* in PubMed, Embase, Web of Science and the Cochrane Library. The accuracy of *GeneXpert vanA/vanB* for *VRE* was evaluated using summary receiver to operate characteristic curve, pooled sensitivity, pooled specificity, positive likelihood ratio, negative likelihood ratio, and diagnostic odds ratio.

**Results:**

8 publications were divided into 3 groups according to two golden standard references, *vanA* and *vanB* group, *vanA* group, *vanB* group, including 6 researches, 5 researches and 5 researches, respectively. The pooled sensitivity and specificity of group *vanA* and *vanB* were 0.96 (95% CI, 0.93–0.98) and 0.90 (95% CI, 0.88–0.91) respectively. The DOR was 440.77 (95% CI, 37.92–5123.55). The pooled sensitivity and specificity of group *vanA* were 0.86 (95% CI, 0.81–0.90) and 0.99 (95% CI, 0.99–0.99) respectively, and those of group *vanB* were 0.85 (95% CI, 0.63–0.97) and 0.82 (95% CI, 0.80–0.83) respectively.

**Conclusion:**

*GeneXpert vanA/vanB* can diagnose *VRE* with high-accuracy and shows greater accuracy in diagnosing *vanA*.

## Author summary

*Vancomycin-resistant enterococci* (VRE), firstly identified in the mid-1980, is a type of antimicrobial resistance bacteria. In recent years, they were found more colonization in patients with critical diseases, showing new resistance to many antibacterial drugs, which is a worrisome clinical problem worldwide. Traditionally, VRE testing is performed mainly by culture which is a standard reference but requires complex steps and takes a long time. Currently, *GeneXpert vanA/vanB* were approved as a rapid and sensitive molecular assay for detecting VRE. However, the accuracy of *GeneXpert vanA/vanB* is without systematic-analyses in evidence-based medicine. Therefore, we conducted a data integration and analysis in this study. Finally, we draw a conclusion that *GeneXpert vanA/vanB* has a high accuracy diagnosing VRE in comparation with conventional culture and PCR. Furthermore, *GeneXpert vanA/vanB* shows more accuracy in diagnosing *vanA*. In addition, we suggest that an additional test is needed for further detecting *vanB*. This finding provides a promising direction for the diagnosis of VRE to a certain extent.

## Introduction

Since 1988, *vancomycin-resistant enterococci* (*VRE*) have been found in patients with critical diseases due to extensive use of antibiotics, prolonged hospital stays and intensive care unit (ICU) admission [1]. They became a type of antimicrobial resistance (*AMR*) bacteria that most commonly spread in medical institutions, especially in Europe [2], with an incidence of 2–34.9% [3]. At present, *VRE* is prevalent globally, and its prevalence has increased significantly, which is a worrisome clinical problem worldwide [4].

*VRE* testing is currently performed mainly by traditional culture and Polymerase Chain Reaction (PCR) detection of the resistance genes *vanA* and *vanB* [5,6]. Although culture is the confirmed reference method [7,8], it takes a long time, requires complex extraction and detection steps and has a high economic impact during a *VRE* outbreak [9]. The U.S. Food and Drug Administration (FDA) approved a rapid molecular assay, the *GeneXpert vanA/vanB* [8,10], which is a unique and completely automated process that includes deoxyribonucleic acid (DNA) extraction, amplification and detection using real-time PCR. Furthermore, results are usually available in less than one hour [4,5].

It is indicated that *GeneXpert vanA/vanB* testing is sensitive as well as cost-effective [5,11]. In addition, there are some researches supporting that some indetermination results exist in that of *GeneXpert vanA/vanB* detecting van B [10,12]. There are few systematic-analyses on the diagnostic accuracy of *GeneXpert vanA/vanB* for *VRE* in evidence-based medicine. Therefore, to appraise the accuracy of *GeneXpert vanA/vanB* in the diagnosis of VRE and distinguish the differences between *GeneXpert vanA/vanB* detecting vanA and vanB, we conducted data integration and analysis.

## Material and methods

### Search strategy

A systematic literature search was carried out for publications until May 03, 2021, related to the diagnostic accuracy of *GeneXpert vanA/vanB* for *VRE*. Four databases were involved: PubMed, Embase, Web of Science and the Cochrane Library. According to PCIO criteria, the search stratagem utilized was as follows: (((*Enterococcus*) AND (*Vancomycin Resistance*)) OR (*Vancomycin-Resistant Enterococci*)) AND (*GeneXpert vanA/vanB*). Possible matches were also retrieved from the related references and the language was restricted to English.

### Study selection

Inclusion criteria:

- Each included study used *GeneXpert VanA/VanB* for detection of *VRE*. Clinical specimens were identified as *VRE* or standard strains by reference methods, which were regarded as the gold standards;
- Human samples were detected and analyzed;
- A 2 × 2 table was constructed with sufficient data to estimate sensitivity, specificity, and the likelihood ratio.

Exclusion criteria:

- Samples from animals or other species;
- Reference standards cannot be found;
- Incomplete raw data: when the raw data were unable to construct the 2 × 2 tables, or when raw data were unable to obtained from the authors;
- Duplicate publications;
- Reviews, conference abstracts, case reports and studies that data extraction was impossible to perform.

Two independent reviewers assessed the studies according to the defined criteria above. If the results were found to be inconsistent, the third investigator was consulted and concluded the same.

### Data extraction

An Excel spreadsheet was created to collect data, which was extracted by two investigators who scanned the included literature independently. Any disagreements were reconciled by a third team member. The following variables comprise the first author’s name, the publication year, the area where the research was implemented, type of study, clinical features and settings, the specimen type, reference standard test, and false and true positives and negatives (TP, TN, FP, FN). When we discuss *vanA* and *vanB* simultaneously, named *vanA* and *vanB* group, Mycobacterial culture was defined as the gold standard. When we discuss *vanA* or *vanB* separately, named *vanA* group and *vanB* group, the golden standard was defined as mycobacterial culture and PCR.

In the studied texts, multiple groups and different backgrounds were considered discrete units of analysis comprising a single study.

### Quality assessment

The quality of the publications were assessed using Quality Assessment of Diagnostic Accuracy Studies (QUADAS-2) [12]. There are four key domains that compose the tool, patient selection, the index test, reference standard and flow and timing, that evaluates bias and utility of the reviewed studies. Values of high, unclear, or low risk were assigned to grade each group of data conducted by different researchers independently figuring out the questions of the four domains. When a divergence appeared, a third investigator was invited to make the final decision.

### Statistical analysis

#### (1) Statistical testing

The pooled sensitivity, specificity, positive likelihood ratios (PLR), negative likelihood ratios (NLR), diagnostic odds ratio (DOR) and 95% confidence intervals (95% CI) were analyzed based on the data provided in the article and evaluated by forest plots, adopting a random- effects model. A value of 0.5 was added to studies with zero values to correct for continuity. A Fagan’s nomogram was facilitated to estimate the clinical application of *GeneXpert vanA/vanB* for the clinical diagnosing of *VRE* [13] by calculating the pre-test and post-test probabilities.

#### (2) Analysis of heterogeneity

In diagnostic experiments, the threshold effect or non-threshold effect might be the primary cause of heterogeneity [14]. We gave priority to ensure whether the threshold effect exists by plotting summary receiver operator characteristic (SROC) curve and further calculating the Spearman correlation coefficient (R). An SROC space shows a typical “shoulder arm” pattern, suggesting the presence of a threshold effect. An R ≥ 0.6 revealed a threshold effect, which manifests a rapid increase of the logit of sensitivity with the logit of 1-specificity adding [15].

Several reasons other than threshold have contributed to the appearance of correlation between sensitivity and specificity [16]. Cochran’s Q test and the inconsistence index (I2) were facilitated to evaluate heterogeneity. When I2<50%, evidence shows no significant heterogeneity, use fixed- effects model. On the contrary, the random- effects model is adopted [17]. We performed meta regression and the sensitivity analysis to investigate potential sources of heterogeneity. AUC (the area under the SROC curve) takes values between 0 and 1, presenting an overall summary performance of studies [18]. To analyze publication bias, Deeks’ funnel plot was applied; P > 0.05 showed that this meta-analysis has no publication bias [19].

#### (3) Tools

Meta-DiSc 1.4 was employed to analyze all data and STATA 12.0 was employed to draw Fagan’s nomogram, bivariate box plot, and evaluating publication bias. Review Manager (RevMan) 5.3 software was applied to conduct the quality assessment.

## Result

### Publications retrieved

There are 53 published studies initially gleaned from the databases Embase (20), Web of Science (18), PubMed (15) and the Cochrane Library (0), of which 24 were left after removing duplicates. According to the titles and abstracts, 8 articles were eliminated. 8 articles were further excluded according to the exclusion criteria, through the full-text review (S1 Fig). Shows the additional reasons for exclusion. Finally, 8 publications [3,5,7,10,11,20,21] satisfied the inclusion criteria. We grouped the involving studies according to two golden standard references, named vanA and vanB group [7,8,10,11,21], vanA group [3,5,7,20,21], vanB group [3,5,7,20,21].

### Description of meta-analyzed publications

Of the 8 articles, the publication years range from 2010 to 2019. Two were from the U.S. Three studies were retrospective while the remaining were prospective. The sample size was comprised 3064 subjects in total, 1563 subjects of which were categorized as vanA and vanB group and 2362 subjects were categorized as vanA group, vanB group. Sample types included rectal swabs, blood cultures, perianal swabs, and stool. Except for the articles which did not refer to the patients, three studies introduced patients from ICUs, one study’s patients suffered from renal dialysis and another’s patients were from hematology or gastroenterology departments. All bacteria were diagnosed as *VRE*.

Study characteristics in Table 1 show individual studies and their characteristics respectively.

**Table 1:** Basic characteristics of included studies [3,5,7,8,10,11,20,21].

Author	Year	Study design	Country	Sample size (No. of patients)	Clinical features and settings	Reference Standard	Specimen type
Both [10]	2019	Retrospective	Germany	33(-)	-^b^	culture	blood cultures
Both [10]	2019	Prospective	Germany	205(-)	-^b^	culture	blood cultures
Marner [11]	2011	Retrospective	America	184(145)	Patients	culture	perianal swabs
Babady [8]	2012	Prospective	America	300(162)	patients in bone marrow transplant units	culture	rectal swabs
Zabicka [21]	2011	Prospective	Poland	37(37)	Patients from Hematology or gastroenterology	1.culture 2. PCR	stool samples
Bourdon [7]	2010	Prospective	France	804(794)	Patients	1.culture 2. PCR	rectal swabs
Goossens [20]	2011	Prospective	Belgium	50(-)	patients undergoing renal dialysis	1.Culture 2.PCR	stool samples
Holzknecht [5]	2017	Prospective	Denmark	1099(804)	patients	1.culture 2. PCR	rectal swabs
Olivgeeris [3]	2016	Retrospective	Greece	372(-)	patients in ICU	1.culture 2.PCR	rectal swabs

a: MIC: minimum inhibitory concentration.

b: No mention of clinical features.

c: No mention of MIC.

### Heterogeneity and publication bias

**Fig 1:**
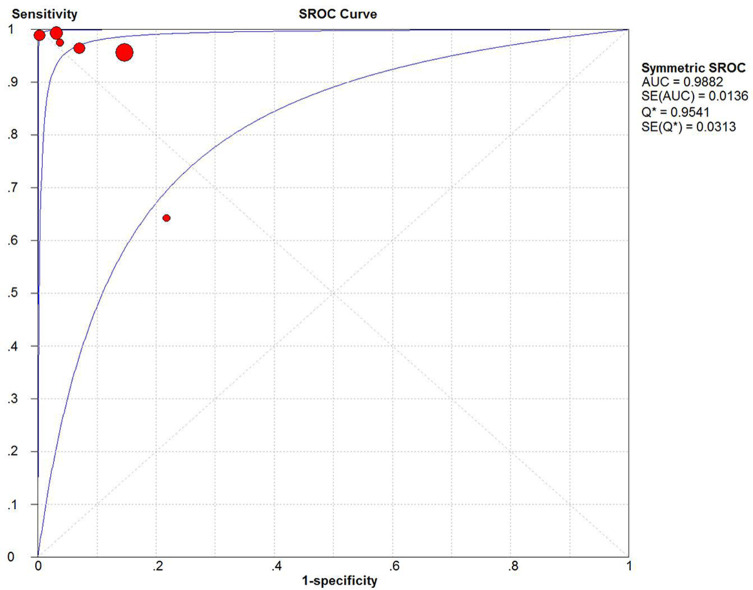
Summary receiver operating curves of vanA and vanB group. The SROC AUC was 0.9882, which is close to 1, indicating a high ability for *VRE* detection.

**Fig 2:**
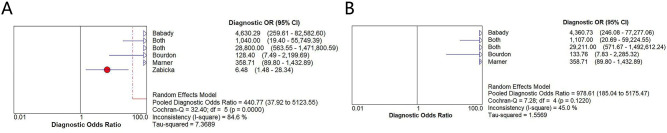
Forest plot for the pooled diagnostic odds ratio of vanA and vanB group. A Forest plot for DOR among 6 studies. B Forest plot for DOR among 5 studies (outlier study was excluded). After excluding the study, the I^2^ value for heterogeneity decreased from 84.6% to 45%.

**Fig 3:**
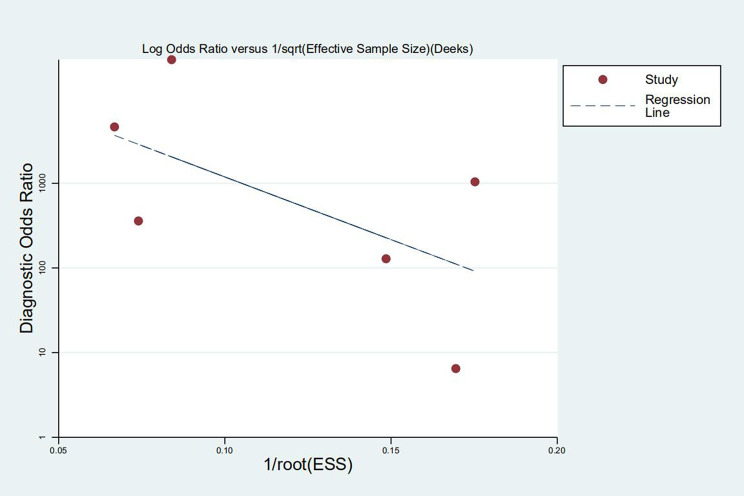
Deeks’ funnel plot asymmetry test of vanA and vanB group. P = 0.279 means no Publication bias.

No “shoulder arm” SROC curve was observed (Fig 1), and the Spearman correlation coefficient (R) was –0.943. In conclusion, there was no evidence of threshold effect. A forest map of DOR (Fig 2A) revealed that Cochran’s Q = 32.40, P ≤ 0.01 and I^2^ = 84.6%, indicating that significant heterogeneity was observed in the included studies. The result of meta regression (Table 2) indicated that sample types might be one possible source of heterogeneity. Sensitivity analysis showed that removal of any study did not alter the significance of the pooled effect size except the study of Zabicka (S2 Fig). After excluding this study, the I^2^ value for heterogeneity decreased to 45% (Fig 2B). According to the method described above, Deeks’ funnel plot showed no substantial asymmetry (P = 0.279). Therefore, publication bias was excluded (Fig 3).

**Table 2:** Possible sources of heterogeneity in the meta-regression analysis.

	Coef	p	95%CI
Specimen type	-1.499	0.023	(-2.615, -0.384)
Study design	0.418	0.609	(-1.919, 2.756)

Coef: Coefficent

### Methodological quality

**Fig 4:**
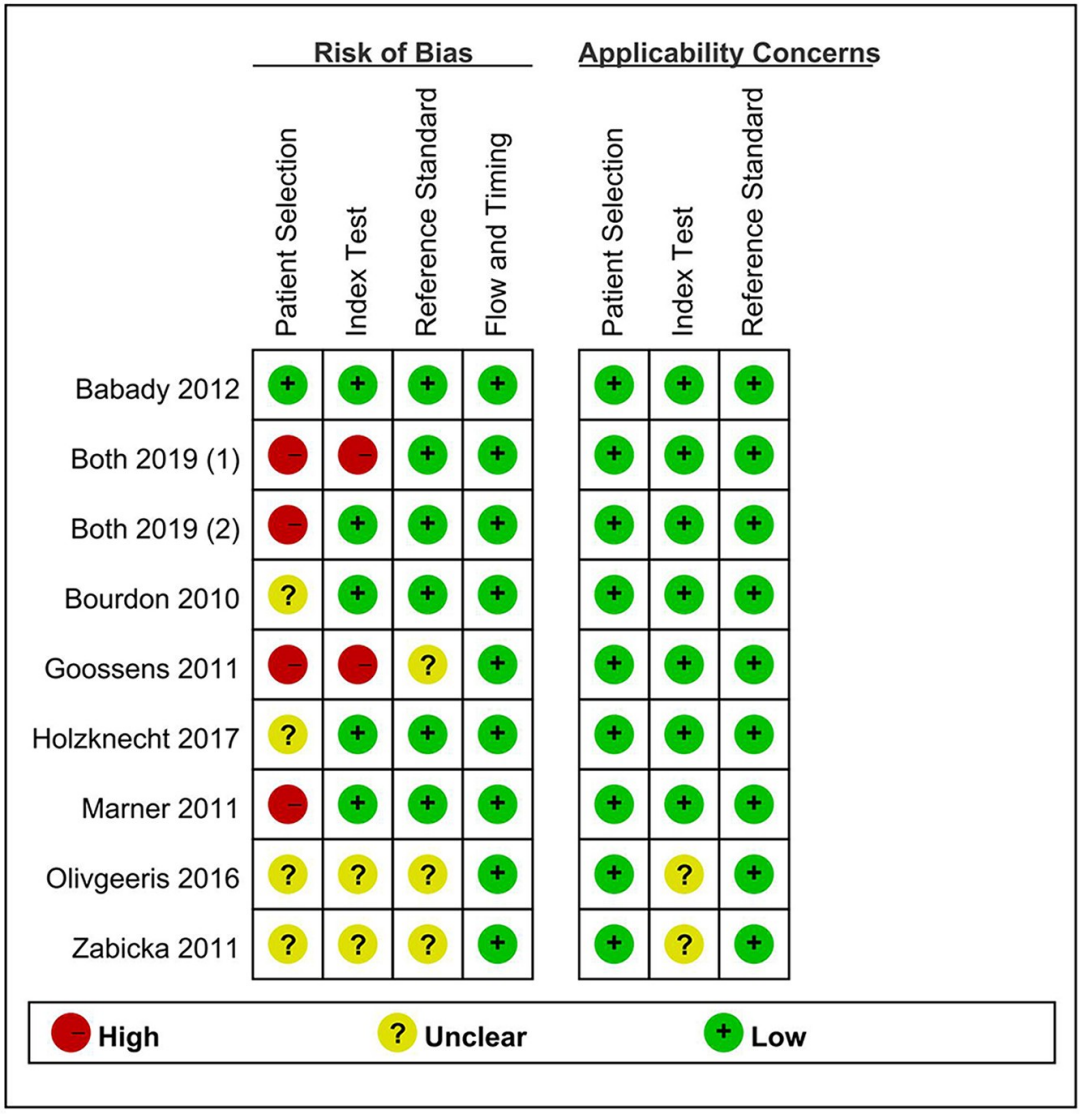
Quality assessment using QUADAS-2 tool for included studies.

Using RevMan 5.3, the overall methodological quality of the included studies is shown in Fig 4. Patient selection and the index test mainly contribute to the risk of bias. In patient selection domain, we assessed four studies as taking a high risk for bias, because they didn’t enroll participants randomly or consecutively, and one had a case-control design [9]. In the field of the index test, two studies were assessed to be high risk for bias: one index test did not use a pre-specified threshold, and the other was explained with prior knowledge of the reference standard results. In the reference standard area, most studies had a low risk of bias, as they stated that the results of the reference standard were interpreted without knowing the index test results. Judging from the index test, the flow and timing of the risk of bias were relatively low. There was no concern about the assessment of applicability for nine studies in the patient selection, the index test and reference standard domain.

### Merge analysis results

**Fig 5:**
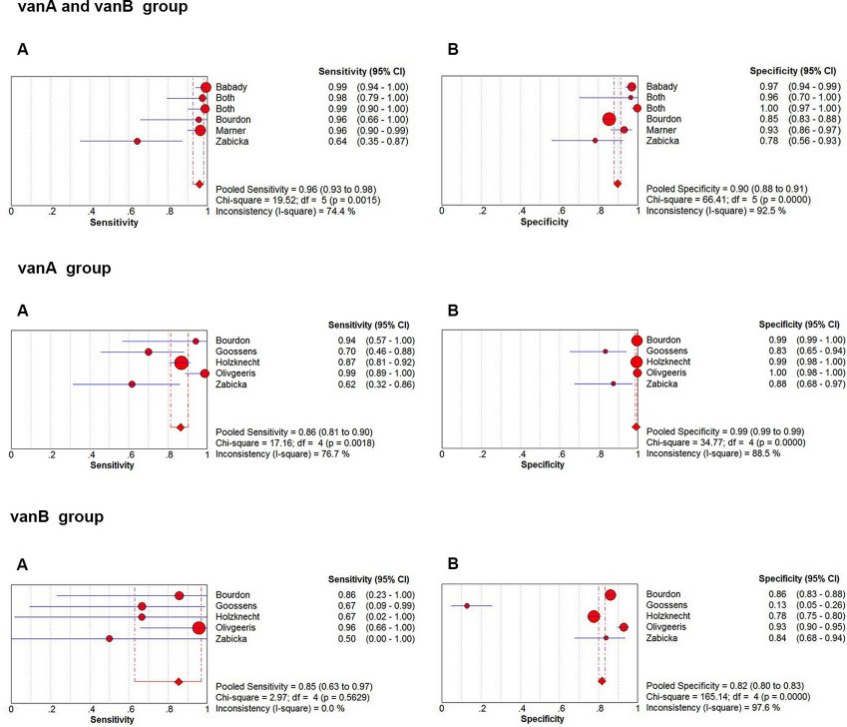
Forest plots for the pooled sensitivity and specificity of three groups. A: sensitivity B: specificity.

The pooled sensitivity, specificity, PLR, NLR and DOR of *GeneXpert VanA/VanB* of each group were shown in Table 3. The pooled sensitivity and specificity were 0.96 (95% CI, 0.93–0.98), 0.90 (95% CI, 0.88–0.91) for vanA and vanB group, 0.86 (95% CI, 0.81–0.90) and 0.99 (95% CI, 0.99–0.99) for vanA group, 0.85 (95% CI, 0.63–0.97) and 0.82 (95% CI, 0.80–0.83) for vanB group, respectively (Fig 5).

**Table 3:** Summarized results of the analysis.

Group	vanA and vanB				vanA				VanB			
	TP*	FP*	FN*	TN*	TP	FP	FN	TN	TP	FP	FN	TN
Babady [8]	74	7	0	219	-	-	-	-	-	-	-	-
Both [10]	20	0	0	13	-	-	-	-	-	-	-	-
Both [10]	45	0	0	160	-	-	-	-	-	-	-	-
Marner [11]	81	7	3	93	-	-	-	-	-	-	-	-
Bourdon [7]	11	116	0	677	8	4	0	792	3	112	0	689
Zabicka [21]	9	5	5	18	8	3	5	21	0	6	0	31
Goossens [20]	-	-	-	-	14	5	6	25	2	41	1	6
Holzknecht [5]	-	-	-	-	145	7	22	925	1	246	0	852
Olivgeeris [3]	-	-	-	-	39	1	0	332	11	26	0	335
Pool sensitivity(95%CI)	0.96(0.93–0.98)				0.86(0.81–0.90)				0.85(0.63–0.97)			
Pool specificity(95%CI)	0.90(0.88–0.91)				0.99(0.99–0.99)				0.82(0.80–0.83)			
PLR (95%CI)	16.44(3.66–73.86)				40.61(6.74–244.53)				3.73(1.15–12.09)			
NLR (95%CI)	0.04(0.00–0.32)				0.18(0.07–0.47)				0.40(0.08–2.16)			
DOR (95%CI)	440.77(37.92–5123.55)				301.18(20.72–4377.94)				10.05(0.77–131.68)			

Legend: -: Data was not provided in articles; TP, true positive; FP, false positive; FN, false negative; TN, true negative; PLR, positive likelihood ratios; NLR, negative likelihood ratios; DOR, diagnostic odds ratio.

**Fig 6:**
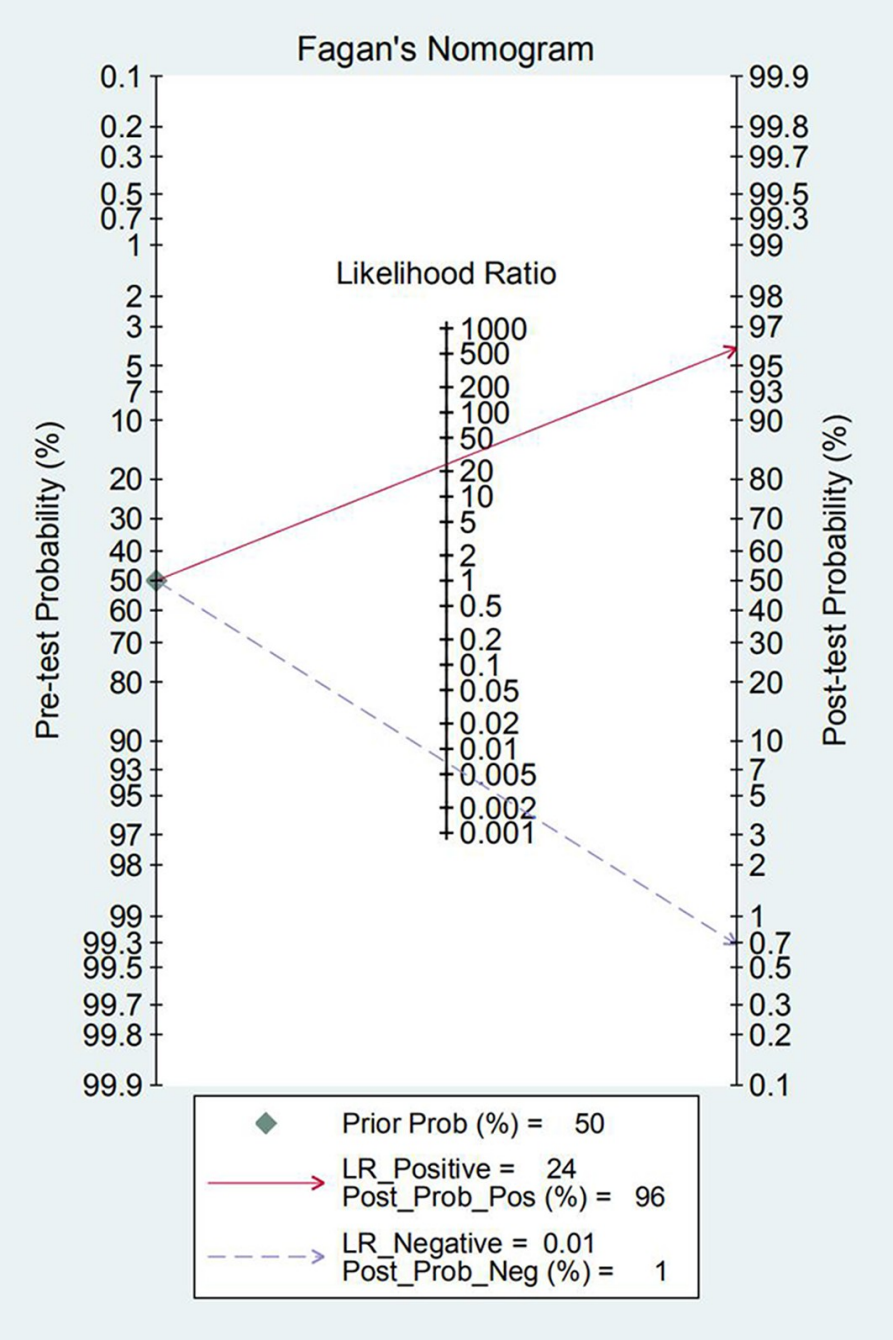
Fagan’s nomogram plot analysis for evaluating clinical application value.

As Fagan’ s nomogram showed, when the pre-test probability was set to 50%, the PLR of the upper diagonal was 24 and the post-test probability was 96%. Correspondingly, the NLR of the lower diagonal was 0.01 and the post-test probability was 1% (Fig 6).

## Discussion

To the best of our knowledge, this is the first meta-analysis accessing the overall diagnostic accuracy of *GeneXpert vanA/vanB*. In this study, we did a thorough search using strict screening criteria, and finally, including 8 articles, groups in different reference standards. The results of our study indicate that *GeneXpert vanA/vanB* assay has a high diagnostic accuracy. Its excellent sensitivity (0.96, 95% CI, 0.93–0.98), specificity (0.90, 95% CI, 0.88–0.91) and DOR (440.77, 95% CI, 37.92–5123.55) made it an attractive option for routine surveillance of *VRE* in the future. The combined PLR and NLR were 16.44 (95%CI, 3.66–73.86) and 0.04 (95%CI, 0.00–0.32), respectively, suggesting that *GeneXpert vanA/vanB* has a brilliant capacity to diagnose and exclude a *VRE*. The SROC AUC was 0.9882, which is close to 1, indicating a high ability for *VRE* detection. Thus, *GeneXpert vanA/vanB* showed a very good diagnostic accuracy. Fagan’s nomogram showed the clinical application value of *GeneXpert vanA/vanB* in various situations.

We also conduct a study on *GeneXpert vanA/vanB* diagnosis discrepancy between *vanA* and *vanB*. The combined sensitivity, specificity, PLR, NLR, DOR of the vanA group were higher than those of the vanB group. Furthermore, the pooled NLR was lower, revealing *GeneXpert vanA/vanB* is more accurate in diagnosis on *vanA*.

That there were more false-positive results in vanB group may be attributed to the presence of genes in several species of aerobic and anaerobic bacteria that were highly similar to the *vanB* sequences [5,7]. It is inevitable for the reason these bacteria also exist in the human [21]. The culture method for all clinical E. faecium isolates may neither be feasible nor cost-efficient in the setting of every routine lab, which makes it impossible to make a clear decision about the need to isolate the patient. Hence, supplementary tests are needed for further investigating [22,23].

Sensitivity analysis demonstrated that the study of Zabicka contributes to heterogeneity. It could be influenced by the factor that the experiment performed during a VanA E. faecium outbreak, as the report of Dekeyser et al. [24], and none of the patients was colonized with VanB enterococci. Several FP vanB results may be concerned with the specimen type, stool swabs. Stool and rectal swabs might be the harbors where anaerobic microbes were commonly checked, which increased the risks of detecting false-positive vanB results [11]. The meta regression also confirmed the specimen types might be one of possible sources of heterogeneity. The discrepancies between *GeneXpert vanA/vanB* detecting vanA and vanB might be a source of heterogeneity. Restrained by only two studies conducting both experiments on vanA and vanB detecting simultaneously or separately, a further analysis is required for more data.

There were still other variables that required to be explored, such as relevant description of patients. The sources and characteristics of patients were quite distinguished. However, sources of heterogeneity could not be formally explored for most tests because few studies were available for further evaluation.

The present study has several limitations. First, remarkable heterogeneity was observed in this study. To account for this heterogeneity, a random effects model was used to synthesis the identified studies in our analysis, which potentially increases the probability of type I error. Moreover, the results of meta regression and the sensitivity analysis were attempted to explain that detected sample could partly explain the source of heterogeneity. Subgroup analysis is looking forward to with more updating data. Second, our study also confirmed the observation of other authors that the *GeneXpert vanA/vanB* test has a low positive predictive value (PPV) for vanB enterococci [25]. Combining additional detection technologies may represent a pragmatic solution to increase VRE detection rates. Finally, we only retrieved published literature from four English databases. Only included studies written in English may have affected our findings. Despite comprehensive searches, the influence of unpublished positive results on the overall results could not be eliminated.

## Conclusion

In summary, *GeneXpert vanA/vanB* has a high accuracy diagnosing *VRE*. Furthermore, *GeneXpert vanA/vanB* shows more accuracy when diagnosing vanA. Additional test is needed for further detecting VanB.

## Supporting information

S1 Fig Flow chart for article search. (PDF)

S2 Fig Sensitivity analysis of each study. Sensitivity analyses showed that removal of any study did not alter the significance of the pooled effect size except the study of Zabicka.
(TIF)

S1 Text PRISMA checklist. (DOC)

## References

[1] Prematunge C, MacDougall C, Johnstone J, Adomako K, Lam F, Robertson J, et al. VRE and VSE Bacteremia Outcomes in the Era of Effective VRE Therapy: A Systematic Review and Meta-analysis. Infect Control Hosp Epidemiol. 2016;37(1):26–35. Epub 2015/10/06. doi: 10.1017/ice.2015.228 ; PubMed Central PMCID: PMC4707508. 26434609 PMC4707508

[2] Mac S, Fitzpatrick T, Johnstone J, Sander B. Vancomycin-resistant enterococci (VRE) screening and isolation in the general medicine ward: a cost-effectiveness analysis. Antimicrob Resist Infect Control. 2019;8:168. Epub 2019/11/07. doi: 10.1186/s13756-019-0628-x ; PubMed Central PMCID: PMC6820905. 31687132 PMC6820905

[3] Papadimitriou-Olivgeris M, Filippidou S, Kolonitsiou F, Drougka E, Koutsileou K, Fligou F, et al. Pitfalls in the identification of Enterococcus species and the detection of vanA and vanB genes. Lett Appl Microbiol. 2016;63(3):189–95. Epub 2016/07/02. doi: 10.1111/lam.12610 . 27367648

[4] Kreidl P, Mayr A, Hinterberger G, Berktold M, Knabl L, Fuchs S, et al. Outbreak report: a nosocomial outbreak of vancomycin resistant enterococci in a solid organ transplant unit. Antimicrob Resist Infect Control. 2018;7:86. Epub 2018/07/24. doi: 10.1186/s13756-018-0374-5 ; PubMed Central PMCID: PMC6052578. 30034798 PMC6052578

[5] Holzknecht BJ, Hansen DS, Nielsen L, Kailow A, Jarløv JO. Screening for vancomycin-resistant enterococci with Xpert® vanA/vanB: diagnostic accuracy and impact on infection control decision making. New Microbes New Infect. 2017;16:54–9. Epub 2017/02/17. doi: 10.1016/j.nmni.2016.12.020 ; PubMed Central PMCID: PMC5295639. 28203378 PMC5295639

[6] Huh HJ, Jang MA, Seo JY, Kim JY, Ki CS, Kim JW, et al. Evaluation of the iNtRON VRE vanA/vanB real-time PCR assay for detection of vancomycin-resistant enterococci. Ann Lab Med. 2015;35(1):76–81. Epub 2015/01/02. doi: 10.3343/alm.2015.35.1.76 ; PubMed Central PMCID: PMC4272969. 25553284 PMC4272969

[7] Bourdon N, Bérenger R, Lepoultier R, Mouet A, Lesteven C, Borgey F, et al. Rapid detection of vancomycin-resistant enterococci from rectal swabs by the Cepheid Xpert vanA/vanB assay. Diagn Microbiol Infect Dis. 2010;67(3):291–3. Epub 2010/06/15. doi: 10.1016/j.diagmicrobio.2010.02.009 . 20542208

[8] Babady NE, Gilhuley K, Cianciminio-Bordelon D, Tang YW. Performance characteristics of the Cepheid Xpert vanA assay for rapid identification of patients at high risk for carriage of vancomycin-resistant Enterococci. J Clin Microbiol. 2012;50(11):3659–63. Epub 2012/09/14. doi: 10.1128/JCM.01776-12 ; PubMed Central PMCID: PMC3486258. 22972822 PMC3486258

[9] Zhou X, Arends JP, Kampinga GA, Ahmad HM, Dijkhuizen B, van Barneveld P, et al. Evaluation of the Xpert vanA/vanB assay using enriched inoculated broths for direct detection of vanB vancomycin-resistant Enterococci. J Clin Microbiol. 2014;52(12):4293–7. Epub 2014/10/10. doi: 10.1128/JCM.01125-14 ; PubMed Central PMCID: PMC4313300. 25297325 PMC4313300

[10] Both A, Berneking L, Berinson B, Lütgehetmann M, Christner M, Aepfelbacher M, et al. Rapid identification of the vanA/vanB resistance determinant in Enterococcus sp. from blood cultures using the Cepheid Xpert vanA/vanB cartridge system. Diagn Microbiol Infect Dis. 2020;96(4):114977. Epub 2020/01/20. doi: 10.1016/j.diagmicrobio.2019.114977 . 31954596

[11] Marner ES, Wolk DM, Carr J, Hewitt C, Dominguez LL, Kovacs T, et al. Diagnostic accuracy of the Cepheid GeneXpert vanA/vanB assay ver. 1.0 to detect the vanA and vanB vancomycin resistance genes in Enterococcus from perianal specimens. Diagn Microbiol Infect Dis. 2011;69(4):382–9. Epub 2011/03/15. doi: 10.1016/j.diagmicrobio.2010.11.005 . 21396533

[12] Whiting PF RA, Westwood ME, Mallett S, Deeks JJ, Reitsma JB, Leeflang MM, Sterne JA, Bossuyt PM; QUADAS-2 Group. QUADAS-2: a revised tool for the quality assessment of diagnostic accuracy studies. Ann Intern Med. 2011;155(8):529–36. doi: 10.7326/0003-4819-155-8-201110180-00009 22007046

[13] Liu CH, Gil-Gómez A, Ampuero J, Romero-Gómez M. Diagnostic accuracy of SCCA and SCCA-IgM for hepatocellular carcinoma: A meta-analysis. Liver Int. 2018;38(10):1820–31. Epub 2018/04/29. doi: 10.1111/liv.13867 . 29704434

[14] Jia X, Li S, Xu T, Ji N, Huang M. Diagnostic accuracy of periostin in predicting asthma: a systematic review and Meta-analysis. J Asthma. 2019:1–9. Epub 2019/11/19. doi: 10.1080/02770903.2019.1684518 . 31738608

[15] Walusimbi S, Bwanga F, De Costa A, Haile M, Joloba M, Hoffner S. Meta-analysis to compare the accuracy of GeneXpert, MODS and the WHO 2007 algorithm for diagnosis of smear-negative pulmonary tuberculosis. BMC Infect Dis. 2013;13:507. Epub 2013/11/01. doi: 10.1186/1471-2334-13-507 ; PubMed Central PMCID: PMC3833313. 24172543 PMC3833313

[16] Zamora J, Abraira V, Muriel A, Khan K, Coomarasamy A. Meta-DiSc: a software for meta-analysis of test accuracy data. BMC Med Res Methodol. 2006;6:31. Epub 2006/07/14. doi: 10.1186/1471-2288-6-31 ; PubMed Central PMCID: PMC1552081. 16836745 PMC1552081

[17] Liu M, Wang SJ, Yang X, Peng H. Diagnostic Efficacy of Sentinel Lymph Node Biopsy in Early Oral Squamous Cell Carcinoma: A Meta-Analysis of 66 Studies. PLoS One. 2017;12(1):e0170322. Epub 2017/01/21. doi: 10.1371/journal.pone.0170322 ; PubMed Central PMCID: PMC5249063. 28107500 PMC5249063

[18] Glas AS, Lijmer JG, Prins MH, Bonsel GJ, Bossuyt PM. The diagnostic odds ratio: a single indicator of test performance. J Clin Epidemiol. 2003;56(11):1129–35. Epub 2003/11/15. doi: 10.1016/s0895-4356(03)00177-x . 14615004

[19] Lee YH, Song GG. Diagnostic accuracy of dual-energy computed tomography in patients with gout: A meta-analysis. Semin Arthritis Rheum. 2017;47(1):95–101. Epub 2017/04/05. doi: 10.1016/j.semarthrit.2017.03.002 . 28372824

[20] Gazin M, Lammens C, Goossens H, Malhotra-Kumar S, Team MWS. Evaluation of GeneOhm VanR and Xpert vanA/vanB molecular assays for the rapid detection of vancomycin-resistant enterococci. Eur J Clin Microbiol Infect Dis. 2012;31(3):273–6. Epub 2011/06/15. doi: 10.1007/s10096-011-1306-y . 21667270

[21] Zabicka D, Strzelecki J, Wozniak A, Strzelecki P, Sadowy E, Kuch A, et al. Efficiency of the Cepheid Xpert vanA/vanB assay for screening of colonization with vancomycin-resistant enterococci during hospital outbreak. Antonie Van Leeuwenhoek. 2012;101(3):671–5. Epub 2011/11/30. doi: 10.1007/s10482-011-9681-z . 22124681

[22] Szymankiewicz M, Wróblewska J, Nowikiewicz T. Incidence of genes encoding vanA/vanB vancomycin resistance in rectal swabs of patients with diagnosed cancer, on the day of admission to hospital, in a non-epidemic period. 2020;15(3):220–4. doi: 10.5114/pg.2020.98537 33005267 PMC7509899

[23] Walker SV, Wolke M, Plum G, Weber RE, Werner G, Hamprecht A. Failure of Vitek2 to reliably detect vanB-mediated vancomycin resistance in Enterococcus faecium. Journal of Antimicrobial Chemotherapy. 2021. doi: 10.1093/jac/dkab101 33855441

[24] Dekeyser S, Beclin E, Descamps D. Intérêt de la mise en place de la recherche des gènes vanA et vanB par technique PCR en système clos (Xpert vanA/vanB Cepheid®) dans un laboratoire de microbiologie dans le cadre de la gestion d’une épidémie à Enterococcus faecium résistant aux glycopeptides (EfRG). Pathologie Biologie. 2011;59(2):73–8. 10.1016/j.patbio.2010.07.013 20828941

[25] Tfifha M, Ferjani A, Mallouli M, Mlika N, Abroug S, Boukadida J. Carriage of multidrug-resistant bacteria among pediatric patients before and during their hospitalization in a tertiary pediatric unit in Tunisia. Libyan J Med. 2018;13(1):1419047-. doi: 10.1080/19932820.2017.1419047 . 29277142 PMC5757224

