# Supplementary figures and images for: Evaluation of GeneXpert vanA/vanB in the early diagnosis of vancomycin-resistant enterococci infection
Source: PLoS Negl Trop Dis. 2021 Nov 8;15(11):e0009869. doi: 10.1371/journal.pntd.0009869 (PMC8575182; doi:10.1371/journal.pntd.0009869)

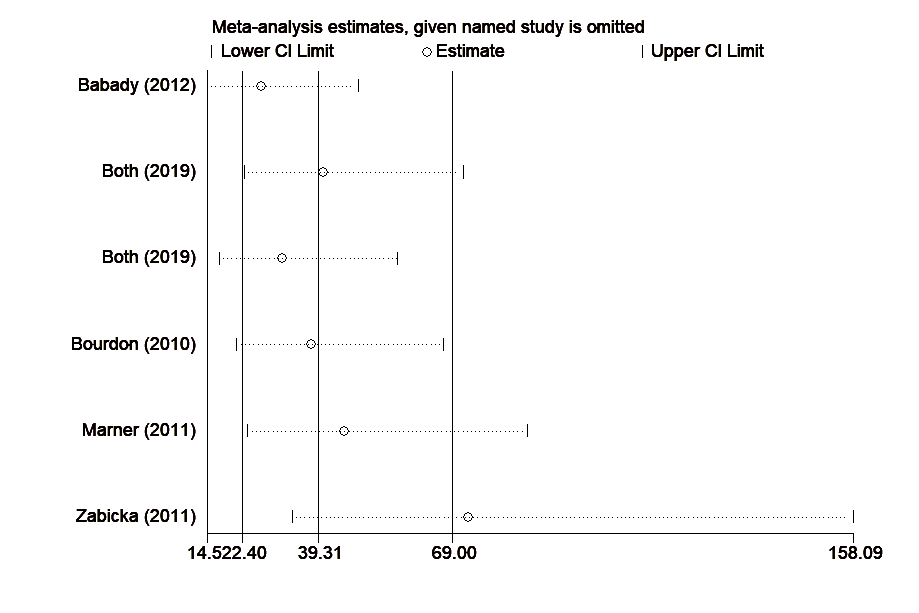

Supplement: S2 Fig — Sensitivity analyses showed that removal of any study did not alter the significance of the pooled effect size except the study of Zabicka. (TIF) [file pntd.0009869.s002.tif]
