# Supplementary material for: Evaluation of GeneXpert vanA/vanB in the early diagnosis of vancomycin-resistant enterococci infection
Source: PLoS Negl Trop Dis. 2021 Nov 8;15(11):e0009869. doi: 10.1371/journal.pntd.0009869 (PMC8575182; doi:10.1371/journal.pntd.0009869)
Supplement: Attachment — Submitted filename: PNTD-D-20-02213_reviewer_04222021.pdf [file pntd.0009869.s004.pdf]

PLOS Neglected Tropical Diseases

Evaluation of GeneXpert vanA/vanB in the early diagnosis of vancomycin-resistant enterococci

--Manuscript Draft--

Manuscript Number:	PNTD-D-20-02213
Full Title:	Evaluation of GeneXpert vanA/vanB in the early diagnosis of vancomycin-resistant enterococci
Short Title:	GeneXpert vanA/vanB in diagnosis of vancomycin-resistant enterococci
Article Type:	Research Article
Keywords:	GeneXpert vanA/vanB; vancomycin-resistant enterococci; culture; diagnose.
Abstract:	Purpose: Vancomycin-resistant enterococci (VRE) infection is a worrying worldwide clinical problem. To evaluate the accuracy of GeneXpert vanA/vanB in the diagnosis of VRE , we conducted a research in the study.<br>Methods: A comprehensive retrieve, review and quality assessment and systematic analysis of publications were performed. The bivariate binomial mixed model measured publication bias, threshold effect and the estimation of diagnostic indicators. Moreover, the sources of heterogeneity were assessed using Cochran's Q test and inconsistency index (I-squared).<br>Results : Eight publications were divided into three groups, vanA and vanB, vanA, vanB, which were included 6 researches, 5 researches and 5 researched, respectively. The pooled sensitivity and specificity of group vanA and vanB were 0.96 (95% CI, 0.93–0.98) and 0.90 (95% CI, 0.88–0.91) respectively. The diagnostic odds ratio (DOR) was 440.77 (95% CI, 37.92–5123.55). The pooled sensitivity and specificity of group vanA were 0.86 (95% CI, 0.81–0.90) and 0.99 (95% CI, 0.99–0.99) respectively. And those of group vanB were 0.85 (95% CI, 0.63–0.97) and 0.82 (95% CI, 0.80–0.83) respectively.<br>Conclusion: GeneXpert vanA/vanB can diagnose VRE with high-precision and shows greater accuracy in diagnosing vanA.
Additional Information:	
Question	Response
**Financial Disclosure**<br><br>Enter a financial disclosure statement that describes the sources of funding for the work included in this submission. Review the submission guidelines for detailed requirements. View published research articles from *PLOS NTDs* for specific examples.<br><br>This statement is required for submission and **will appear in the published article** if the submission is accepted. Please make sure it is accurate.	The author(s) received no specific funding for this work.

Powered by Editorial Manager® and ProduXion Manager® from Aries Systems Corporation

**Unfunded studies**<br><br>Enter: *The author(s) received no specific funding for this work.*<br><br>**Funded studies**<br><br>Enter a statement with the following details:<br>• Initials of the authors who received each award<br>• Grant numbers awarded to each author<br>• The full name of each funder<br>• URL of each funder website<br>• Did the sponsors or funders play any role in the study design, data collection and analysis, decision to publish, or preparation of the manuscript?<br>• **NO** - Include this sentence at the end of your statement: *The funders had no role in study design, data collection and analysis, decision to publish, or preparation of the manuscript.*<br>• **YES** - Specify the role(s) played.<br><br>* typeset	
**Competing Interests**<br><br>Use the instructions below to enter a competing interest statement for this submission. On behalf of all authors, disclose any competing interests that could be perceived to bias this work—acknowledging all financial support and any other relevant financial or non-financial competing interests.<br><br>This statement **will appear in the published article** if the submission is accepted. Please make sure it is accurate. View published research articles from *PLOS NTDs* for specific examples.	The authors have declared that no competing interests exist.

**NO authors have competing interests**<br><br>Enter: *The authors have declared that no competing interests exist.*<br><br>**Authors with competing interests**<br><br>Enter competing interest details beginning with this statement:<br><br>*I have read the journal's policy and the authors of this manuscript have the following competing interests: [insert competing interests here]*<br><br>* typeset	
**Data Availability**<br><br>Authors are required to make all data underlying the findings described fully available, without restriction, and from the time of publication. PLOS allows rare exceptions to address legal and ethical concerns. See the PLOS Data Policy and FAQ for detailed information.<br><br>A Data Availability Statement describing where the data can be found is required at submission. Your answers to this question constitute the Data Availability Statement and **will be published in the article**, if accepted.	Yes - all data are fully available without restriction

Powered by Editorial Manager® and ProduXion Manager® from Aries Systems Corporation

**Important:** Stating 'data available on request from the author' is not sufficient. If your data are only available upon request, select 'No' for the first question and explain your exceptional situation in the text box.<br><br>Do the authors confirm that all data underlying the findings described in their manuscript are fully available without restriction?	
**Describe where the data may be found in full sentences. If you are copying our sample text, replace any instances of XXX with the appropriate details.**<br><br>• If the data are **held or will be held in a public repository**, include URLs, accession numbers or DOIs. If this information will only be available after acceptance, indicate this by ticking the box below. For example: *All XXX files are available from the XXX database (accession number(s) XXX, XXX.*).<br>• If the data are all contained **within the manuscript and/or Supporting Information files**, enter the following: *All relevant data are within the manuscript and its Supporting Information files.*<br>• If neither of these applies but you are able to provide **details of access elsewhere**, with or without limitations, please do so. For example:<br><br>*Data cannot be shared publicly because of [XXX]. Data are available from the XXX Institutional Data Access / Ethics Committee (contact via XXX) for researchers who meet the criteria for access to confidential data.*<br><br>*The data underlying the results presented in the study are available*	All files are available from Pubmed, Embase and Web of Science.

Powered by Editorial Manager® and ProduXion Manager® from Aries Systems Corporation

from (include the name of the third party and contact information or URL).<br>• This text is appropriate if the data are owned by a third party and authors do not have permission to share the data.<br><br>* typeset	
Additional data availability information:	Tick here if the URLs/accession numbers/DOIs will be available only after acceptance of the manuscript for publication so that we can ensure their inclusion before publication.

Powered by Editorial Manager® and ProduXion Manager® from Aries Systems Corporation

Manuscript Click here to access/download;Manuscript;Manuscript .docx

Evaluation of *GeneXpert vanA/vanB* in the early diagnosis of vancomycin-resistant enterococci

Zhuo-Lei Li[1,2], Qi-Bing Luo[1,2], Shan-Shan Xiao[1,2], Ze-Hong Lin[1,3], Ye-Ling Liu[1,4], Meng-Yi Han[1,4], Jing-Hua Zhong[1,4], Tian-Xing Ji[5], Xu-Guang Guo[1,4,6,7*].

[1] Department of Clinical Laboratory Medicine, The Third Affiliated Hospital of Guangzhou Medical University, Guangzhou, 510150, China;

[2] Department of Clinical Medicine, The Second Clinical School of Guangzhou Medical University, Guangzhou, 511436, China;

[3] Department of Pharmacy, Guangzhou Medical University School of Pharmaceutical Sciences, Guangzhou, 511436, China;

[4] Department of Clinical Medicine, The Third Clinical School of Guangzhou Medical University, Guangzhou, 511436, China;

[5] Department of Clinical Laboratory Medicine, The Second Affiliated Hospital of Guangzhou Medical University, 510260, China;

[6] Key Laboratory for Major Obstetric Diseases of Guangdong Province, The Third Affiliated Hospital of Guangzhou Medical University, Guangzhou, 510150, China;

[7] Key Laboratory for Major Obstetric Diseases of Guangdong Province, The Third Affiliated Hospital of Guangzhou Medical University, Guangzhou, 510150, China;

***** Corresponding author

E-mail: gysygxg@gmail.com (XGG)

Abstract

Purpose: *Vancomycin-resistant enterococci* (*VRE*) infection is a worrying worldwide clinical problem. To evaluate the accuracy of *GeneXpert vanA/vanB* in the diagnosis of *VRE*, we conducted a research in the study.

Methods: A comprehensive retrieve, review and quality assessment and systematic analysis of publications were performed. The bivariate binomial mixed model measured publication bias, threshold effect and the estimation of diagnostic indicators. Moreover, the sources of heterogeneity were assessed using Cochran's Q test and inconsistency index (I-squared).

Results: Eight publications were divided into three groups, vanA and vanB, vanA, vanB, which were included 6 researches, 5 researches and 5 researched, respectively. The pooled sensitivity and specificity of group vanA and vanB were 0.96 (95% CI, 0.93–0.98) and 0.90 (95% CI, 0.88–0.91) respectively. The diagnostic odds ratio (DOR) was 440.77 (95% CI, 37.92–5123.55). The pooled sensitivity and specificity of group vanA were 0.86 (95% CI, 0.81–0.90) and 0.99 (95% CI, 0.99–0.99) respectively. And those of group vanB were 0.85 (95% CI, 0.63–0.97) and 0.82 (95% CI, 0.80–0.83) respectively.

Conclusion: *GeneXpert vanA/vanB* can diagnose *VRE* with high-precision and shows greater accuracy in diagnosing vanA.

Keywords： GeneXpert vanA/vanB; vancomycin-resistant enterococci; culture; diagnose.

Introduction

Since 1988, *vancomycin-resistant enterococci* (*VRE*) have been found in patients with critical diseases due to extensive use of antibiotics, prolonged hospital stay and intensive care unit (ICU) admission [1]. They became a type of antimicrobial resistance (*AMR*) bacteria that most commonly spread in medical institutions, especially in Europe [2], with an incidence of 2–34.9% [4]. At present, *VRE* is prevalent globally, and its prevalence has increased significantly, which is a worrying clinical problem worldwide [5].

VRE testing is currently performed mainly by traditional culture and Polymerase Chain Reaction (PCR) detection of the resistance genes vanA and vanB [6,7]. Although culture is a confirmed reference method [8,9], it takes a long time, requires complex extraction and detection steps and has a high economic impact during a *VRE* outbreak [10]. The U.S. Food and Drug Administration (FDA) approved a rapid molecular assay, the *GeneXpert vanA/vanB* [9,11], which is a unique and completely automated process that includes deoxyribonucleic acid (DNA) extraction, real-time PCR amplification and detection. Furthermore, results are usually available in less than an hour [5,6]. Thus, it is confirmed that *GeneXpert vanA/vanB* testing is a sensitive as well as cost-effective method as compared to the traditional culture [6,12]. However, the overall diagnostic precision of *GeneXpert vanA/vanB* for *VRE* remains unclear. Hence, to appraise the accuracy of *GeneXpert vanA/vanB* in the diagnosis of *VRE* and discriminate the differences between *GeneXpert vanA/vanB* detecting vanA and vanB, we conducted this research.

Material and Methods

Search strategy

A systematic literature search was carried out for publications until July 15, 2020, related to the diagnostic accuracy of *GeneXpert vanA/vanB* for *VRE*. Four databases were involved: PubMed, Embase, Web of Science and the Cochrane Library. The search stratagem utilized was as follows: (((*Enterococcus*) AND (*Vancomycin Resistance*)) OR (*Vancomycin-Resistant Enterococci*)) AND (*GeneXpert vanA/vanB*). Possible matches were also retrieved from the related references and the language was restricted to English.

Study selection

Inclusion criteria:

(i) Each included study used *GeneXpert VanA/VanB* for the detection of *VRE*. Clinical specimens were identified as *VRE* or standard strains by reference methods, which were regarded as the gold standards;

(ii) Humans samples were detected and analyzed;

(iii) A 2 × 2 table was constructed with sufficient data to estimate sensitivity, specificity and likelihood ratio.

Exclusion criteria:

(i) Samples taken from animals or other species;

(ii) When reference standards cannot be found;

(iii) Incomplete raw data, when the raw data was unable to construct the 2 × 2 tables, or when we failed to associate authors to obtain raw data;

(iv) Some duplicate publications which were published repeatedly;

(v) Reviews, conference abstracts, case reports and studies that do not allow data extraction.

Two independent reviewers assessed the studies according to the above-defined criteria. If the results were found to be inconsistent, the third investigator was

consulted and concluded the same.

Data extraction

We created an Excel spreadsheet to collect data, extracted by two investigators who screened the whole texts of included literature independently. The following variables comprise the first author's last name, publication year, the area where the research was implemented, type of study, the duration when it was conducted, clinical features and settings, sample size, specimen type, reference standard test, minimum inhibitory concentration (MIC) and false and true positives and negatives (TP, TN, FP, FN) In the studied texts, multiple groups and different backgrounds were considered discrete units of analysis comprising a single study.

Quality assessment

The included publications were assessed for quality using Quality Assessment of Diagnostic Accuracy Studies (QUADAS-2) [13]. There are four key domains that constitute the tool, patient selection, index test, reference standard and flow and timing, that evaluates bias and utility of the reviewed studies. Values of high, unclear or low risk were assigned to grade each group of data conducted by different researchers independently figuring out the questions of the four domains. When a divergence appeared, a third expert investigator was invited to join to determine the answers.

Statistical analysis

(1) Statistical testing

The pooled sensitivity, specificity, positive likelihood ratios (PLR), negative likelihood ratios (NLR), diagnostic odds ratio (DOR) and 95% confidence intervals (95% CI) were analyzed based on the data provided in the article and evaluated by

forest plots, adopting a random-effects model. A value of 0.5 was added to studies with zero values to correct for continuity. A Fagan's nomogram was facilitated to estimate the clinical application of *GeneXpert vanA/vanB* for the clinical diagnosing of *VRE* [14] by calculating the pre-test and post-test probabilities.

(2) Analysis of heterogeneity

In diagnostic experiments, the threshold effect or non-threshold effect might be the primary cause of heterogeneity [15].We gave priority to ensure whether the threshold effect exists by plotting summary receiver operator characteristic (SROC) curve and further calculating the Spearman correlation coefficient (R). An $R \geq 0.6$ revealed a threshold effect, which manifests a rapid increase of the logit of sensitivity with the logit of 1-specificity adding [16].

Several reasons other than threshold contribute to the appearance of correlation between sensitivity and specificity [17]. Cochran's Q test and the inconsistence index (I-squared) were facilitated to evaluate the heterogeneity [18].An I-squared higher than 50% and a P-value less than 0.05 for the Q test indicated the existence of substantial interstudy heterogeneity [18]. We also made use of a bivariate box plot to illustrate the degree of deviation of each study [15].

AUC (the area under the SROC curve) takes values between 0 and 1, presenting an overall summary performance of studies [19].

To analyze publication bias, Deeks' funnel plot was applied; $P > 0.05$ showed that this meta-analysis has no publication bias [20].

(3) Tools

Meta-DiSc 1.4 was employed to analyze all data and STATA 12.0 was employed to draw Fagan's nomogram, bivariate box plot, and evaluating publication bias. Review Manager (RevMan) 5.3 software was applied to conduct the quality assessment.

Result

Publications retrieved

Forty-five published studies were initially gleaned from the databases Embase (18), Web of Science (15), PubMed (12) and the Cochrane Library (0), of which 24 were removed as they were duplicates. According to the titles and abstracts, eight articles were eliminated. Furthermore, we excluded five articles according to the exclusion criteria, through the full-text review (S1 Fig. shows the additional reasons for the exclusion). Finally, eight publications [4, 6, 8, 9, 11, 12, 21, 22] satisfied the inclusion criteria. We dividing the involving studies into two types according vanA and vanB detected simultaneously or separately, which were comprised of five articles respectively, totally extracted three groups of summary datasets, named vanA and vanB group [8, 9, 11, 12, 22] , vanA group [4, 6, 8, 21, 22], vanB group [4, 6, 8, 21, 22].

Description of meta-analyzed publications

Of the eight articles, the publication years ranged from 2010 to 2019. Six of the eight publications were conducted in European countries including Germany, France, Poland, Belgium, Denmark, Greece. Two were from the U.S. Three studies were retrospective while the remaining were prospective. The sample size was totally comprised 3064 subjects, 1563 subjects of which was categorized as vanA and vanB group and 2362 subjects of which was categorized as vanA group, vanB group. Sample types included rectal swabs, blood cultures, perianal swabs and stool samples. Except for the articles which did not refer to the patients, three studies introduced patients that were from ICUs, one study's patients suffered from renal dialysis and another's patients were referred from hematology or gastroenterology departments. The studies involved reference standard tests like a combination of culture with or

without enriched broth, PCR and clinical chart review. All bacteria were diagnosed as *VRE*.

Study characteristics in Table 1 show individual studies and their characteristics respectively.

Table 1. Basic characteristics of included studies [9,11,8,21,6,12,4,22].

Reference standard test	MIC（minimum inhibitory concentration）
1.traditional culture<br>2.Enriched broth culture<br>3.real-time PCR	vanA: a vancomycin MIC of ＞32μg/ml and a teicoplanin MIC of ＞32 μg/ml<br>vanB: a vancomycin MIC of ＞32μg/ml and a teicoplanin MIC of ＜32 μg/ml
1.sub-cultures by MALDI-TOF<br>2.*VRE* selective agar	-
1.sub-cultures by MALDI-TOF<br>2.*VRE* selective agar	-
1.culture<br>2.the vancomycin-teicoplanin E-test, subculture on agar plate<br>3. Multiplex PCR	-
1.Culture<br>2.PCR and sequencing	-
culture results combined with information from the patients earlier and later samples	vanA: a vancomycin MIC of ≥256 mg/L and a teicoplanin MIC of 8-256 mg/L<br>vanB: a vancomycin MIC of 128 mg/L and a teicoplanin MIC of 0.5 mg/L
the combination of direct agar plating, broth-enriched culture, and clinical chart review.	a vancomycin MIC value of ＞32 μg/mL
ChromID *VRE* combined with in-house PCR	van A: a vancomycin MIC_{50} of 256 mg /L and a teicoplanin MIC_{50} of 24 mg/L<br>vanB: a vancomycin MIC_{50} of 4 mg /L and a teicoplanin MIC_{50} of 0.5 mg/L
The culture after enrichment	a vancomycin MIC value＞256mg/L

Year	Study design	Countries	Duration	Clinical features and settings	Specimen type	Sample size (No. of patients)
2012	Prospective	USA	-	patients in intensive care and bone marrow transplant units	Consecutive rectal swabs	300(162)
2019	Retrospective	Germany	2018.9-2018.11	-	blood cultures	33(-)
2019	Prospective	Germany	2018.12-2019.9	-	blood cultures	205(-)
2010	Prospective	France	2008.4-2008.7	-	rectal swabs	804(794)
2011	Prospective	Belgium	-	patients undergoing renal dialysis	stool samples	50(-)
2017	Prospective	Denmark	2014.12.30-2015.8.15	24 different departments from four different hospitals	rectal swabs	1099(804)
2011	Retrospective	USA	-	①Bone marrow transplant ward (29.5%) ②adult ICU (23.8%) ③pediatric ICU (7%) ④other inpatient wards (12.6) ⑤outpatient setting (4.2%） ⑥no ward location（22.9%）	perianal swab specimens	184(145)
2016	Greece	Retrospective	-	ICU patients	rectal samples	372(-)
2011	Prospective	Poland	2009 3.31-2009 4.17	①hematology (29 patients) ② gastroenterology (8 patients)	stool samples	37(37)

**Authors**	Babady [9]	Both [11]	Both [11]	Bourdon [8]	Goossens [21]	Holzknecht [6]	Marner [12]	Olivgeeris [4]	Zabicka [22]

Legend：-: characteristic was not provided in articles.

Heterogeneity and publication bias

An SROC space shows a typical “shoulder arm” pattern, indicating increasing sensitivities and rapidly decreasing specificities, suggesting the presence of a threshold effect [23]. We did not find this pattern in this study’s SROC curve (Fig 1). Moreover, the Spearman correlation coefficient (R) was –0.943. In conclusion, there was no evidence of the threshold effect. A forest map, which was used to draw the ratio, revealed that Cochran’s $Q = 32.40$, $P \leq 0.01$ and I-squared = 84.6%, indicating that significant heterogeneity was observed in the included studies. The bivariate box plot (Fig 2) showed that one study by Babady et al. [9] fell outside the bivariate box plot, which might cause heterogeneity. According to the method described above, Deeks’ funnel plot showed no substantial asymmetry ($P = 0.279$). Therefore, publication bias was excluded (Fig 3).

Fig 1: Summary receiver operating curves of vanA and vanB group.

Fig 2 The bivariate box plot of vanA and vanB group. ①: study by Babady et al; ②: study by Both et al; ③: another study by Both et al； ④: study by Bourdon et al; ⑤: study by Marner et al; ⑥: study by Zabicka et al.

Fig 3. Deeks’ funnel plot asymmetry test of vanA and vanB group.

Methodological quality

Using RevMan 5.3, the overall methodological quality of the included studies is showed in Fig 4. Patient selection and the index test mainly contribute to the risk of bias. In patient selection domain, we assessed four studies as having a high risk for bias, because they didn't enroll participants randomly or consecutively, and one had a case-control design [10]. Due to a lack of information surrounding the selection process, four of nine studies were considered to have an unclear risk of bias. In the field of the index test, two studies were assessed to be high risk for bias: one's index test did not use a pre-specified threshold, and the other was explained with prior knowledge of the reference standard results. Also, due to an inadequate description, two studies were judged to be at unclear risk of bias. In the reference standard area, most studies had a low risk of bias, as they stated that the results of the reference standard were interpreted without knowing the index test results. Judging from the index test, the flow and timing of the risk of bias were relatively low. There was no concern about the assessment of applicability for nine studies in the patient selection, index test and reference standard domain. Finally, in two studies, the description of the index test was not clear.

Fig 4. Quality assessment using QUADAS-2 tool for included studies.

Merge analysis results

GeneXpert VanA/VanB was employed to detect *VRE* pooled sensitivity, specificity, PLR, NLR and DOR, whose values of each groups were shown in Table 2. The pooled sensitivity and specificity were 0.96 (95% CI, 0.93–0.98), 0.90 (95% CI, 0.88–0.91) for vanA and vanB group, 0.86 (95% CI, 0.81–0.90) and 0.99 (95% CI, 0.99–0.99) for vanA group, 0.85 (95% CI, 0.63–0.97) and0.82 (95% CI, 0.80–0.83) for vanB group, respectively (Fig 5).

As Fagan’ s nomogram showed, when we set the pre-test probability to 50%, the PLR of the upper diagonal was 24 and the post-test probability was 96%. Correspondingly, the NLR of the lower diagonal was 0.01and the post-test probability was 1% (Fig 6).

Table 2. Summarized results of the analysis.

Group	vanA and vanB				vanA				vanB			
	TP	FP	FN	TN	TP	FP	FN	TN	TP	FP	FN	TN
Babady [9]	74	7	0	219	-	-	-	-	-	-	-	-
Both [11]	20	0	0	13	-	-	-	-	-	-	-	-
Both [11]	45	0	0	160	-	-	-	-	-	-	-	-
Marner [12]	81	7	3	93	-	-	-	-	-	-	-	-
Bourdon [8]	11	116	0	677	8	4	0	792	3	112	0	689
Zabicka [22]	9	5	5	18	8	3	5	21	0	6	0	31
Goossens [21]	-	-	-	-	14	5	6	25	2	41	1	6
Holzknecht [6]	-	-	-	-	145	7	22	925	1	246	0	852
Olivgeeris [4]	-	-	-	-	39	1	0	332	11	26	0	335
Pool sensitivity(95%CI)	0.96(0.93-0.98)				0.86(0.81-0.90)				0.85(0.63-0.97)			
Pool specificity(95%CI)	0.90(0.88-0.91)				0.99(0.99-0.99)				0.82(0.80-0.83)			
PLR (95%CI)	16.44(3.66-73.86)				40.61(6.74-244.53)				3.73(1.15-12.09)			
NLR (95%CI)	0.04(0.00-0.32)				0.18(0.07-0.47)				0.40(0.08-2.16)			
DOR (95%CI)	440.77(37.92-5123.55)				301.18(20.72-4377.94)				10.05(0.77-131.68)			

Legend: -: Data was not provided in articles; TP, true positive; FP, false positive; FN, false negative; TN, true negative; PLR, positive likelihood ratios; NLR, negative likelihood ratios; DOR, diagnostic odds ratio.

Fig 5. Forest plots for the pooled sensitivity and specificity of three groups. A: sensitivity B: specificity.

Fig 6. Fagan’s nomogram plot analysis for evaluating clinical application value.

Discussion

It’s essential to identify *VRE* accurately and rapidly for managing and treating both colonized and infected patients as well as preventing the spread of this resistant bacteria in hospitals.

To the best of our knowledge, this is the first meta-analysis conducted to access the overall diagnostic accuracy of *GeneXpert vanA/vanB* for *VRE*. In this study, we did a thorough search using strict screening criteria, and finally, eight articles were included. The results of our study indicate that the *GeneXpert vanA/vanB* assay has [illegible] high diagnostic accuracy, its excellent merge sensitivity (0.96, 95% CI, 0.93–0.98), specificity (0.90, 95% CI, 0.93–0.98) and DOR (440.77, 95% CI, 37.92–5123.55) made it an attractive option for routine surveillance of *VRE* in the future. The combined PLR and NLR were 16.44(95%CI, 3.66–73.86) and 0.04(95%CI, 0.00–0.32), respectively, concluding that *GeneXpert vanA/vanB* has a brilliant capacity to diagnose and exclude a *VRE* diagnosis. The SROC AUC was 0.9882, which is close to 1, indicating a high ability for *VRE* detection. Thus, *GeneXpert vanA/vanB* showed a very good diagnostic accuracy with respect to *VRE*. Fagan’s nomogram showed the clinical application value of *GeneXpert vanA/vanB* in different situations.

The combined sensitivity, specificity, PLR, NLR, DOR of the vanA group were higher than those of the vanB group. Furthermore, the pooled NLR was lower, revealing *GeneXpert vanA/vanB* performing a more precise diagnosis on vanA. This explains why there were more false-positive results in the vanB group which were attributed to the presence of genes in several species of aerobic and anaerobic bacteria that were highly similar to the vanB sequences [6, 8].This is important as these bacteria also exist in the human body [22]. Additionally, the pooled specificity (0.99, 95%CI, 0.99–0.99) of the vanA group increased when compared with that of the vanA and vanB group (0.90, 95%CI, 0.88–0.91), suggesting that the discrepancies between *GeneXpert*

vanA/vanB detecting vanA and vanB might be a source of the studies' heterogeneity. There were still other variables that required study, such as time, sample type, the number of samples, relevant description of patients and the choice of the reference standard and the vancomycin MIC. Our study was comprised of stool, rectal swabs, perianal swabs and blood cultures. Stool and rectal swabs might be the harbors where anaerobic microbes were commonly checked, which increased the risks for detecting false-positive vanB results [12]. The number of samples was 37–804 and the patients' sources and characteristics were quite distinguished. Moreover, the vancomycin MIC has influences on the sensitivity of reference standard [24]. However, sources of heterogeneity could not be formally explored for most tests because few studies were available for further evaluation.

The present study has several limitations. First, we only retrieved published literature from four English databases. Despite comprehensive searches, the influence of unpublished positive results on the overall results could not be eliminated. Additionally, the significant impact of parameters in studies such as the age, sources and clinical features of patients, the duration of the experiments and vancomycin MIC on the pooled values was uncertain and their quality was not well illustrated. Finally, due to the element of data reports of the tested samples, the small sample size, the inadequate description and the limited comparability, group evaluation could not be conducted.

Conclusion

In summary, *GeneXpert vanA/vanB* has a high accuracy when diagnosing *VRE* with high pooled sensitivity (0.96, 95%CI, 0.93–0.98) and high pooled specificity (0.90, 95%CI, 0.88–0.91). Furthermore, *GeneXpert vanA/vanB* shows discrepancies between detecting vanA and vanB in that it shows more accuracy when diagnosing van A.

Funding

This research did not receive any specific grant from funding agencies in the public, commercial, or not-for-profit sectors.

Acknowledgement

Not applicable.

Authors' contributions

Conceptualization and Methodology, ZL Li, YL Liu and XG Guo. Validation, ZL Li and TX Ji. Formal analysis and investigation, ZL Li, QB Luo, SS Xiao and ZH Lin. Writing-Original Draft, ZL Li and QB Luo. Writing-Review& Editing, SS Xiao, ZH Lin, MY Han and JH Zhong. Visualization, ZL Li and QB Luo. Supervision, TX Ji and XG Guo.

References

1. Prematunge C, MacDougall C, Johnstone J, Adomako K, Lam F, Robertson J, et al. *VRE* and VSE Bacteremia Outcomes in the Era of Effective *VRE* Therapy: A Systematic Review and Meta-analysis. Infect Control Hosp Epidemiol. 2016;37(1):26-35. Epub 2015/10/06. doi: 10.1017/ice.2015.228. PubMed PMID: 26434609; PubMed Central PMCID: PMCPMC4707508.

2. Mac S, Fitzpatrick T, Johnstone J, Sander B. Vancomycin-resistant enterococci (*VRE*) screening and isolation in the general medicine ward: a cost-effectiveness analysis. Antimicrob Resist Infect Control. 2019;8:168. Epub 2019/11/07. doi: 10.1186/s13756-019-0628-x. PubMed PMID: 31687132; PubMed Central PMCID: PMCPMC6820905.

3. Cetinkaya Y, Falk P, Mayhall CG. Vancomycin-resistant enterococci. Clin Microbiol Rev. 2000;13(4):686-707. Epub 2000/10/12. doi: 10.1128/cmr.13.4.686-707.2000. PubMed PMID: 11023964; PubMed Central PMCID: PMCPMC88957.

4. Papadimitriou-Olivgeris M, Filippidou S, Kolonitsiou F, Drougka E, Koutsileou K, Fligou F, et al. Pitfalls in the identification of Enterococcus species and the detection of vanA and vanB genes. Lett Appl Microbiol. 2016;63(3):189-95. Epub 2016/07/02. doi: 10.1111/lam.12610. PubMed PMID: 27367648.

5. Kreidl P, Mayr A, Hinterberger G, Berktold M, Knabl L, Fuchs S, et al. Outbreak report: a nosocomial outbreak of vancomycin resistant enterococci in a solid organ transplant unit. Antimicrob Resist Infect Control. 2018;7:86. Epub 2018/07/24. doi: 10.1186/s13756-018-0374-5. PubMed PMID: 30034798; PubMed Central PMCID: PMCPMC6052578.

6. Holzknecht BJ, Hansen DS, Nielsen L, Kailow A, Jarløv JO. Screening for vancomycin-resistant enterococci with Xpert® vanA/vanB: diagnostic accuracy and

impact on infection control decision making. New Microbes New Infect. 2017;16:54-9. Epub 2017/02/17. doi: 10.1016/j.nmni.2016.12.020. PubMed PMID: 28203378; PubMed Central PMCID: PMCPMC5295639.

7. Huh HJ, Jang MA, Seo JY, Kim JY, Ki CS, Kim JW, et al. Evaluation of the iNtRON *VRE* vanA/vanB real-time PCR assay for detection of vancomycin-resistant enterococci. Ann Lab Med. 2015;35(1):76-81. Epub 2015/01/02. doi: 10.3343/alm.2015.35.1.76. PubMed PMID: 25553284; PubMed Central PMCID: PMCPMC4272969.

8. Bourdon N, Bérenger R, Lepoultier R, Mouet A, Lesteven C, Borgey F, et al. Rapid detection of vancomycin-resistant enterococci from rectal swabs by the Cepheid Xpert vanA/vanB assay. Diagn Microbiol Infect Dis. 2010;67(3):291-3. Epub 2010/06/15. doi: 10.1016/j.diagmicrobio.2010.02.009. PubMed PMID: 20542208.

9. Babady NE, Gilhuley K, Cianciminio-Bordelon D, Tang YW. Performance characteristics of the Cepheid Xpert vanA assay for rapid identification of patients at high risk for carriage of vancomycin-resistant Enterococci. J Clin Microbiol. 2012;50(11):3659-63. Epub 2012/09/14. doi: 10.1128/jcm.01776-12. PubMed PMID: 22972822; PubMed Central PMCID: PMCPMC3486258.

10. Zhou X, Arends JP, Kampinga GA, Ahmad HM, Dijkhuizen B, van Barneveld P, et al. Evaluation of the Xpert vanA/vanB assay using enriched inoculated broths for direct detection of vanB vancomycin-resistant Enterococci. J Clin Microbiol. 2014;52(12):4293-7. Epub 2014/10/10. doi: 10.1128/jcm.01125-14. PubMed PMID: 25297325; PubMed Central PMCID: PMCPMC4313300.

11. Both A, Berneking L, Berinson B, Lütgehetmann M, Christner M, Aepfelbacher M, et al. Rapid identification of the vanA/vanB resistance determinant in Enterococcus sp. from blood cultures using the Cepheid Xpert vanA/vanB cartridge

system. Diagn Microbiol Infect Dis. 2020;96(4):114977. Epub 2020/01/20. doi: 10.1016/j.diagmicrobio.2019.114977. PubMed PMID: 31954596.

12. Marner ES, Wolk DM, Carr J, Hewitt C, Dominguez LL, Kovacs T, et al. Diagnostic accuracy of the Cepheid GeneXpert vanA/vanB assay ver. 1.0 to detect the vanA and vanB vancomycin resistance genes in Enterococcus from perianal specimens. Diagn Microbiol Infect Dis. 2011;69(4):382-9. Epub 2011/03/15. doi: 10.1016/j.diagmicrobio.2010.11.005. PubMed PMID: 21396533.

13. Whiting PF RA, Westwood ME, Mallett S, Deeks JJ, Reitsma JB, Leeflang MM, Sterne JA, Bossuyt PM; QUADAS-2 Group. QUADAS-2: a revised tool for the quality assessment of diagnostic accuracy studies. Ann Intern Med. 2011;155(8):529-36. doi: 10.7326/0003-4819-155-8-201110180-00009.

14. Liu CH, Gil-Gómez A, Ampuero J, Romero-Gómez M. Diagnostic accuracy of SCCA and SCCA-IgM for hepatocellular carcinoma: A meta-analysis. Liver Int. 2018;38(10):1820-31. Epub 2018/04/29. doi: 10.1111/liv.13867. PubMed PMID: 29704434.

15. Jia X, Li S, Xu T, Ji N, Huang M. Diagnostic accuracy of periostin in predicting asthma: a systematic review and Meta-analysis. J Asthma. 2019:1-9. Epub 2019/11/19. doi: 10.1080/02770903.2019.1684518. PubMed PMID: 31738608.

16. Walusimbi S, Bwanga F, De Costa A, Haile M, Joloba M, Hoffner S. Meta-analysis to compare the accuracy of GeneXpert, MODS and the WHO 2007 algorithm for diagnosis of smear-negative pulmonary tuberculosis. BMC Infect Dis. 2013;13:507. Epub 2013/11/01. doi: 10.1186/1471-2334-13-507. PubMed PMID: 24172543; PubMed Central PMCID: PMCPMC3833313.

17. Zamora J, Abraira V, Muriel A, Khan K, Coomarasamy A. Meta-DiSc: a software for meta-analysis of test accuracy data. BMC Med Res Methodol. 2006;6:31. Epub

2006/07/14. doi: 10.1186/1471-2288-6-31. PubMed PMID: 16836745; PubMed Central PMCID: PMCPMC1552081.

18. Liu M, Wang SJ, Yang X, Peng H. Diagnostic Efficacy of Sentinel Lymph Node Biopsy in Early Oral Squamous Cell Carcinoma: A Meta-Analysis of 66 Studies. PLoS One. 2017;12(1):e0170322. Epub 2017/01/21. doi: 10.1371/journal.pone.0170322. PubMed PMID: 28107500; PubMed Central PMCID: PMCPMC5249063.

19. Glas AS, Lijmer JG, Prins MH, Bonsel GJ, Bossuyt PM. The diagnostic odds ratio: a single indicator of test performance. J Clin Epidemiol. 2003;56(11):1129-35. Epub 2003/11/15. doi: 10.1016/s0895-4356(03)00177-x. PubMed PMID: 14615004.

20. Lee YH, Song GG. Diagnostic accuracy of dual-energy computed tomography in patients with gout: A meta-analysis. Semin Arthritis Rheum. 2017;47(1):95-101. Epub 2017/04/05. doi: 10.1016/j.semarthrit.2017.03.002. PubMed PMID: 28372824.

21. Gazin M, Lammens C, Goossens H, Malhotra-Kumar S, Team MWS. Evaluation of GeneOhm VanR and Xpert vanA/vanB molecular assays for the rapid detection of vancomycin-resistant enterococci. Eur J Clin Microbiol Infect Dis. 2012;31(3):273-6. Epub 2011/06/15. doi: 10.1007/s10096-011-1306-y. PubMed PMID: 21667270.

22. Zabicka D, Strzelecki J, Wozniak A, Strzelecki P, Sadowy E, Kuch A, et al. Efficiency of the Cepheid Xpert vanA/vanB assay for screening of colonization with vancomycin-resistant enterococci during hospital outbreak. Antonie Van Leeuwenhoek. 2012;101(3):671-5. Epub 2011/11/30. doi: 10.1007/s10482-011-9681-z. PubMed PMID: 22124681.

23. Kang SK, Zhang A, Pandharipande PV, Chandarana H, Braithwaite RS, Littenberg B. DWI for Renal Mass Characterization: Systematic Review and Meta-Analysis of Diagnostic Test Performance. AJR Am J Roentgenol. 2015;205(2):317-

24. Epub 2015/07/24. doi: 10.2214/ajr.14.13930. PubMed PMID: 26204281.

24. Wijesuriya TM, Perry P, Pryce T, Boehm J, Kay I, Flexman J, et al. Low vancomycin MICs and fecal densities reduce the sensitivity of screening methods for vancomycin resistance in Enterococci. J Clin Microbiol. 2014;52(8):2829-33. Epub 2014/05/30. doi: 10.1128/jcm.00021-14. PubMed PMID: 24871216; PubMed Central PMCID: PMCPMC4136124.

Supporting information

S1 Fig. Flow chart for article search.

S2 Text. PRISMA checklist.

S3 Text. CRediT author statement.

Tables and figures' legends

Table 1. Basic characteristics of included studies.

Table 2. Summarized results of the analysis.

Fig 1. Summary receiver operating curves of vanA and vanB group.

Fig 2. The bivariate box plot of vanA and vanB group.

Legend: ①: study by Babady et al; ②: study by Both et al; ③: another study by Both et al； ④: study by Bourdon et al; ⑤: study by Marner et al; ⑥: study by Zabicka et al.

Fig 3. Deeks' funnel plot asymmetry test of vanA and vanB group.

Fig 4. Quality assessment using QUADAS-2 tool for included studies.

Fig 5. Forest plots for the pooled sensitivity and specificity of three groups.

Legend: A: sensitivity B: specificity.

Fig 6. Fagan's nomogram plot analysis for evaluating clinical application value.

Click here to access/download;Figure;Fig1.tif

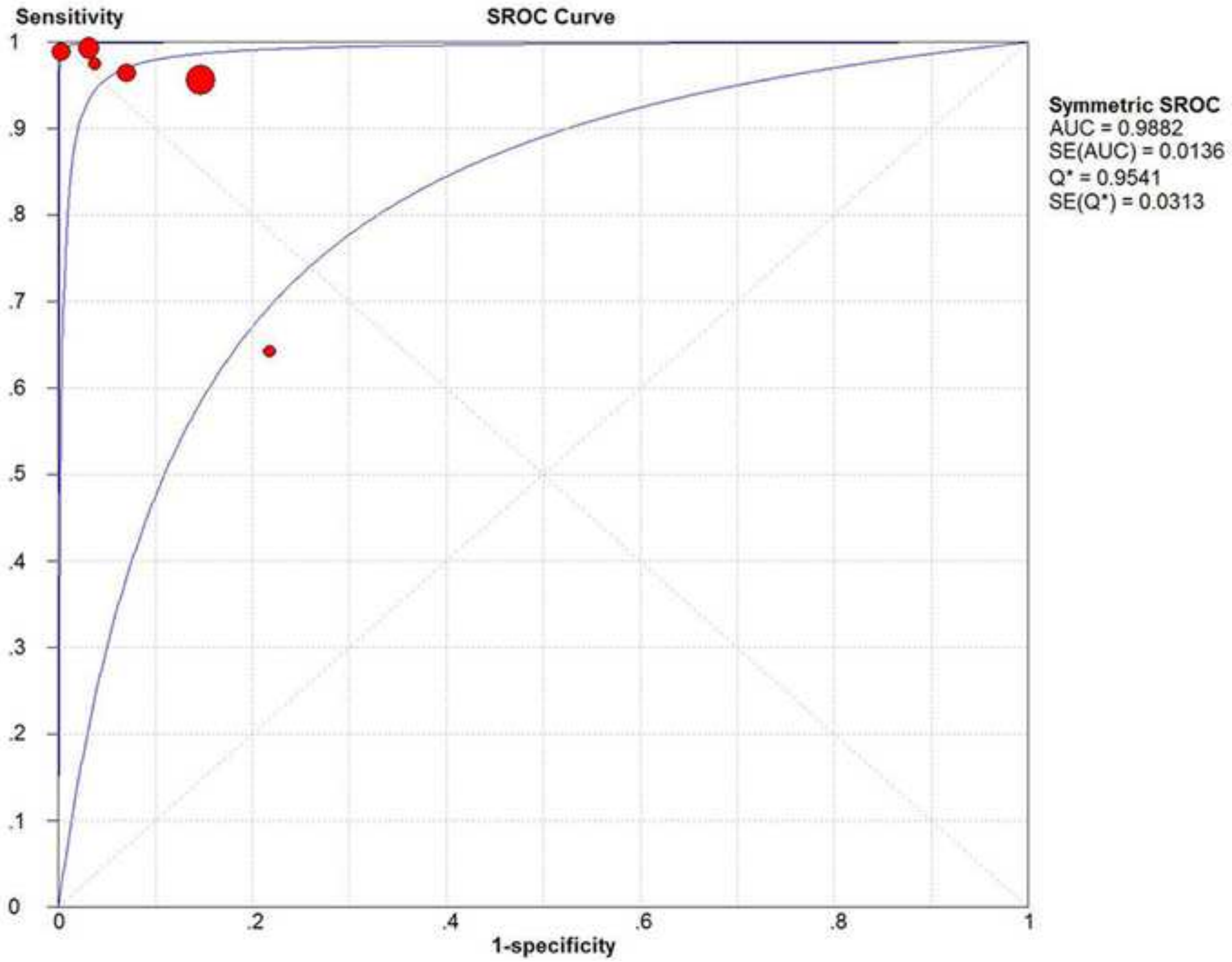

Click here to access/download;Figure;Fig2.tif

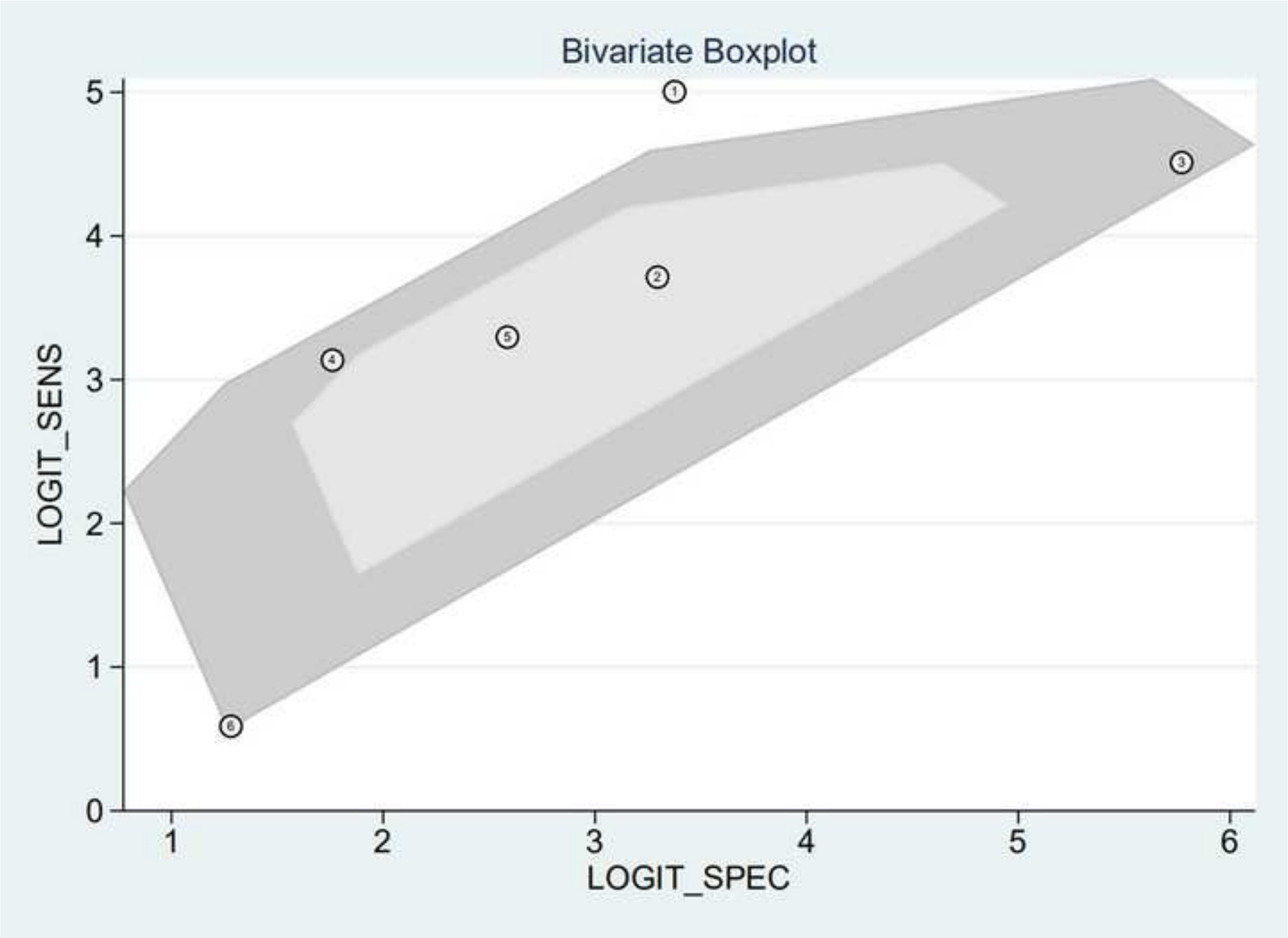

Click here to access/download;Figure;Fig3 .tif

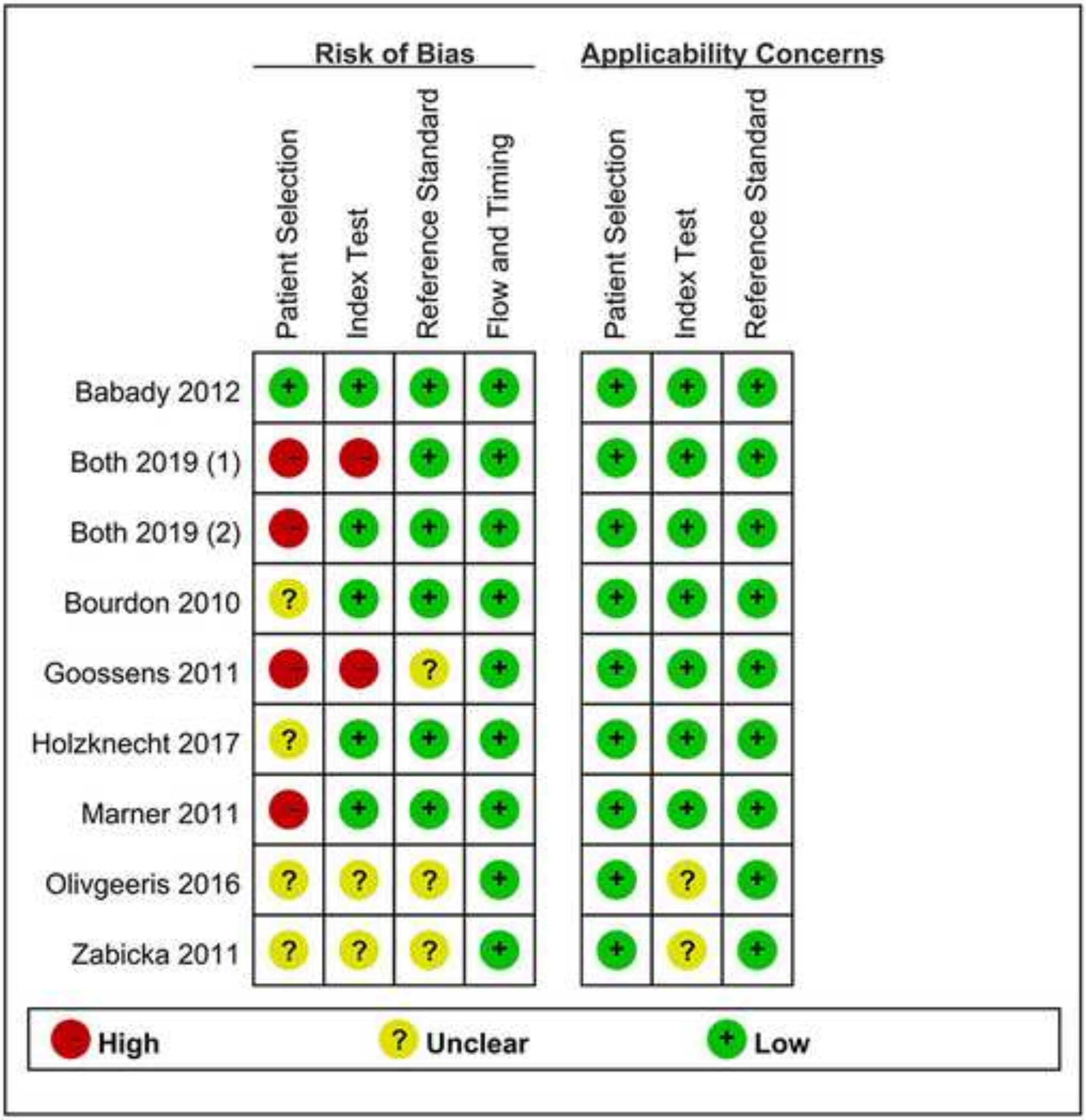

Click here to access/download;Figure;Fig4 .tif

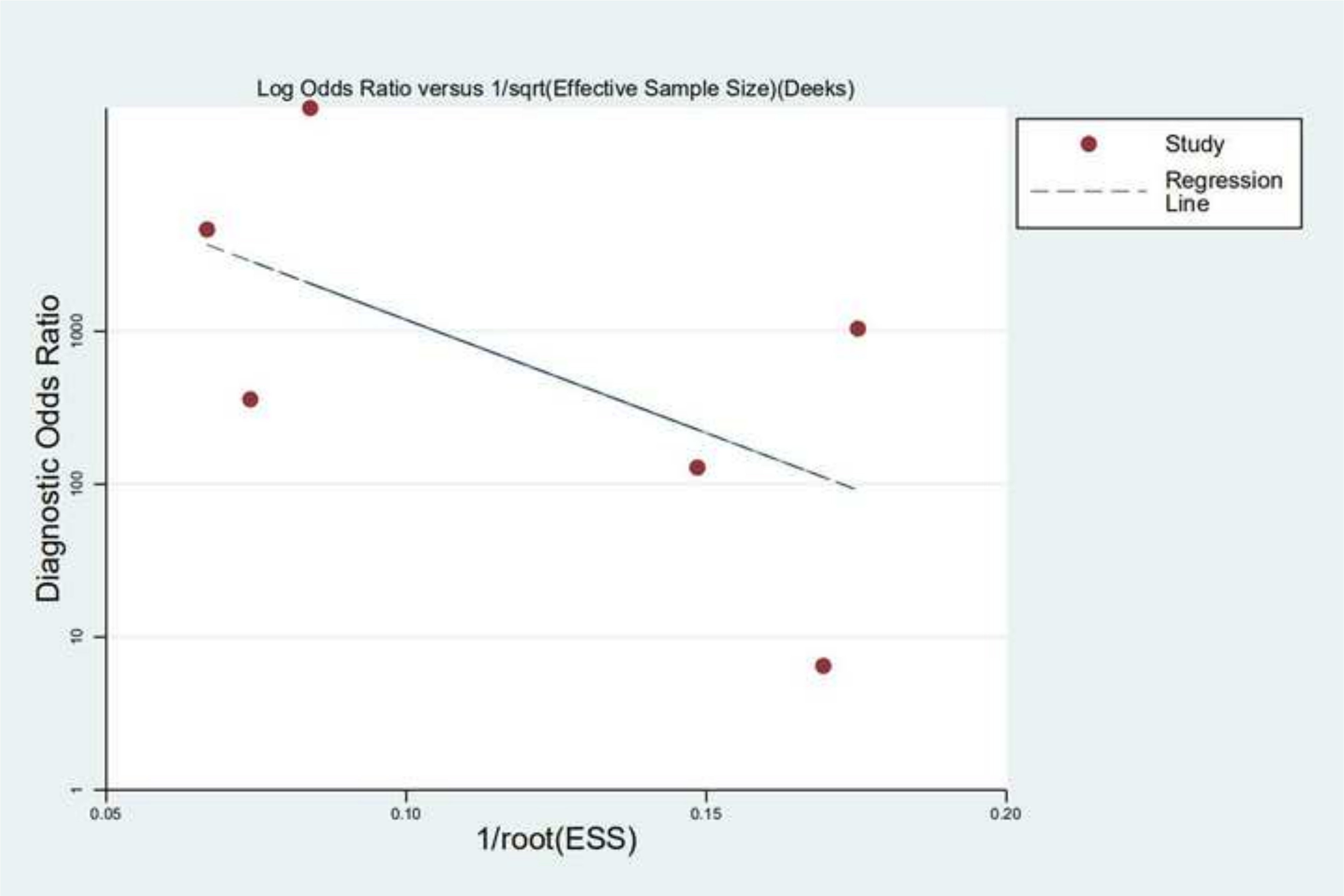

Click here to access/download;Figure;Fig5.tif

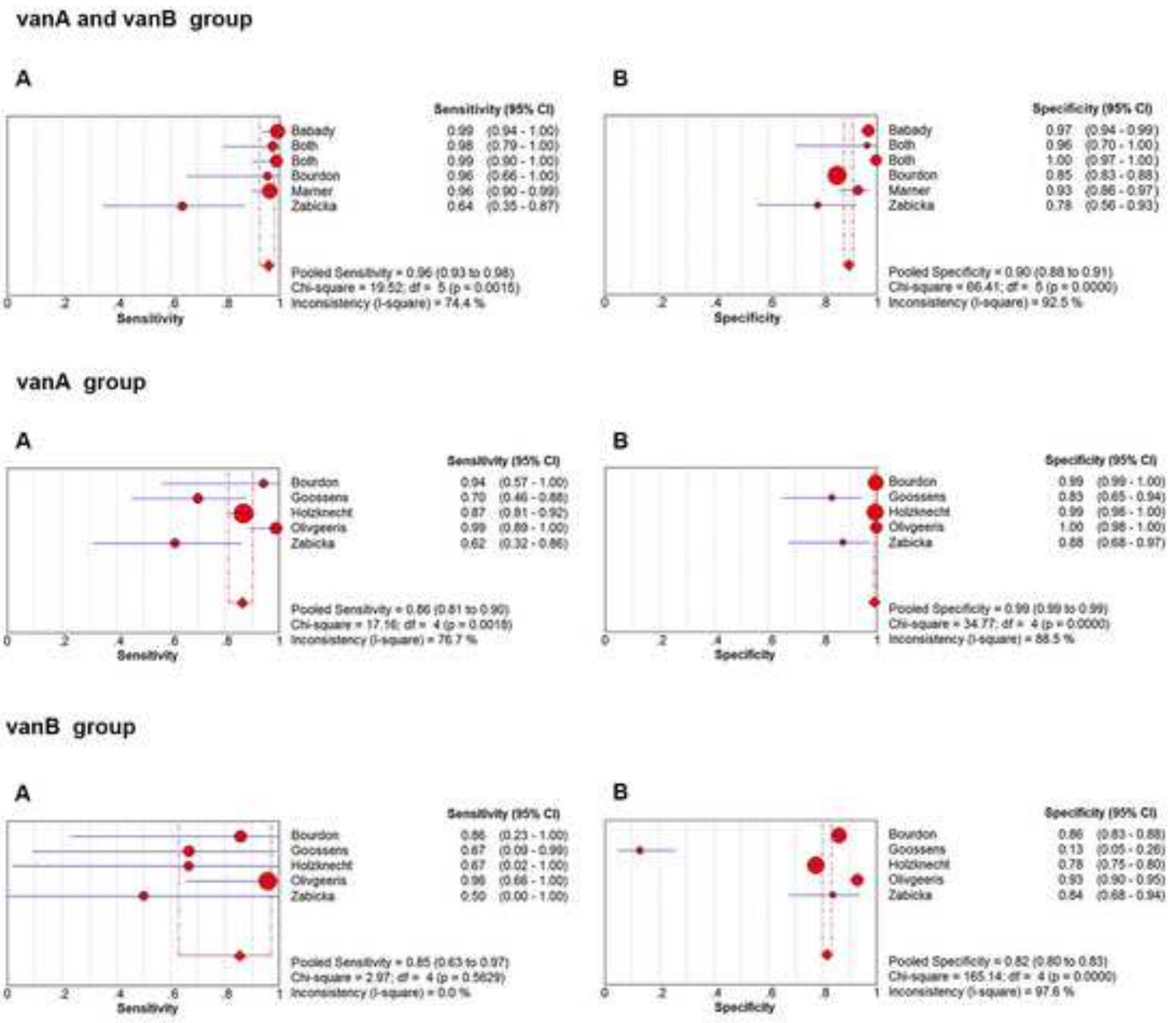

Click here to access/download;Figure;Fig6.tif

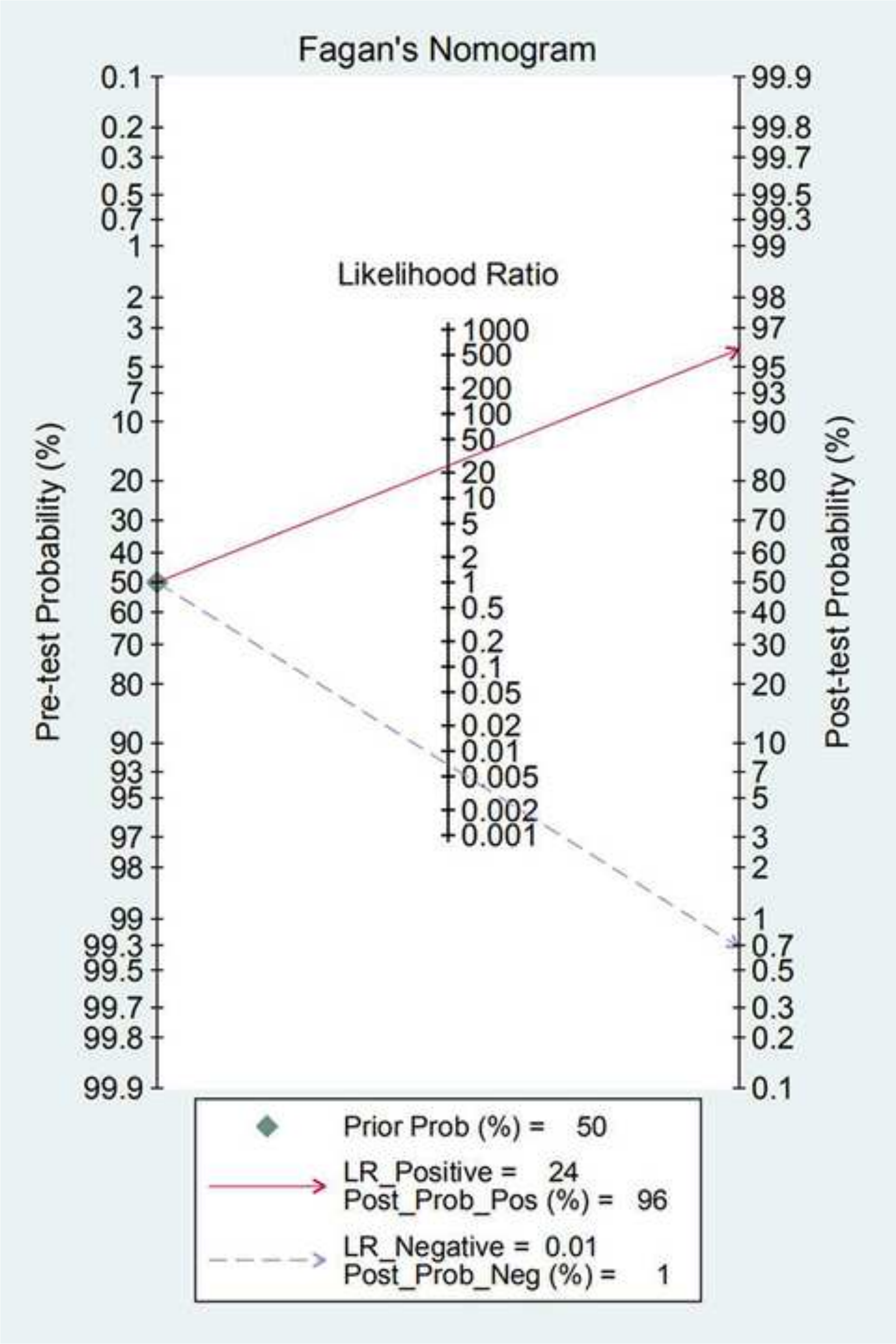

Click here to access/download
Supporting Information
S1 Fig.pdf

Click here to access/download

Supporting Information

S2 Text.doc

Click here to access/download
Supporting Information
S3 Text.doc
