# Supplementary material for: Evaluation of GeneXpert vanA/vanB in the early diagnosis of vancomycin-resistant enterococci infection
Source: PLoS Negl Trop Dis. 2021 Nov 8;15(11):e0009869. doi: 10.1371/journal.pntd.0009869 (PMC8575182; doi:10.1371/journal.pntd.0009869)
Supplement: Attachment — Submitted filename: Response to Reviewers.docx [file pntd.0009869.s005.docx]

Dear Dr. Hasegawa,

Sincerest thanks for your response and reviewers’ comments on our manuscript entitled "*Evaluation of GeneXpert vanA/vanB in the early diagnosis of vancomycin-resistant enterococci*" (Manuscript number: PNTD-S-20-02757). We have revised the manuscript, and would like to re-submit it for your consideration. We have addressed the comments raised by the reviewers, and the amendments are highlighted in yellow in the revised manuscript. Point by point responses to the reviewers’ comments are listed below this letter.

We hope that the revised version of the manuscript is now acceptable for publication in your journal.

I am looking forward to hearing from you soon.

With best wishes,

Yours sincerely,

Xu-Guang Guo.

We would like to express our sincere thanks to the reviewers for the constructive and positive comments.

Replies to Reviewer 1

Specific Comments

**① The study is based on a literature review with no explanation as to how the raw data (if used) was acquired.**

Response：

Thank you for this valuable feedback. The reference standards were clarified explicitly again, which is complemented in Line 105-109, as shown below. Literature will be excluded without following the standard. According to inclusion criteria and exclusion criteria, the articles we studies were original researches whose raw data is shown in detail as results. In the case of raw data can’t be acquired, some measures to associate authors will be taken to obtain the raw data such as taking a phone or sending e-mails. In the absence of raw data, judged by exclusion criteria, the article will be excluded, that is why 3 articles did not be included, shown by S1 Fig. In data extraction, two reviewers scanned the whole texts of included literature independently. Once the result of *GeneXpert vanA/vanB* was consistent with reference standard’s, which we defined as TP, we will categorize it as one of TP. If a different opinion was found between two reviewers, a third investigator would consult and drew conclusions together.

As the details had showed in Line 105-109, “When we discuss vanA and vanB simultaneously, named vanA and vanB group, we define the golden standard reference as mycobacterial culture. When we discuss vanA or vanB separately, named vanA group and vanB group, we define the golden standard reference as mycobacterial culture and the result of PCR.”

**② Based on the bias's and the rejection criteria the sample size is at best 2 studies, not only is this is not a sufficient sample size the techniques used by the researchers of these studies vary introducing significant bias in the analysis presented in this paper.**

Response:

Thank you very much for your constructive comments. As the question that the reviewer had pointed out, we seriously consider whether our selection process is reasonable once again.

We carried out a systematic literature search once more for publications. Finally, 8 articles were still maintained to analysis, evaluated the study’s eligibility based on inclusion/exclusion criteria, and assessed the methodological quality of the studies using the quality assessment of diagnostic accuracy studies (QUADAS-2) checklist again. It is our fault that we didn’t paid enough attention to the risk of bias. Therefore, we supplementary meta regression and sensitivity analysis to investigate potential sources of heterogeneity. Subgroup analysis is looking forward more updating data. The results of sensitivity analysis show that one study have an effect on heterogeneity. After omitting it, the heterogeneity decreased from 87% to 45% (<50%). We also discussed reasons of study causing heterogeneity. By means of meta regression, we investigate one of potential sources of heterogeneity was specimen type.

As the details had showed in Line 175-181, “A forest map of DOR (Fig 2A), revealed that Cochran’s Q = 32.40, P ≤ 0.01 and I-squared = 84.6%, indicating that significant heterogeneity was observed in the included studies. The result of meta regression (Table 2) indicate that sample types might be one of possible sources of heterogeneity. Sensitivity analyses showed that removal of any study did not alter the significance of the pooled effect size except the study of Zabicka^[21]^. After excluding the study, the I^2^ value for heterogeneity decreased to 45% (Fig 2B)”.

Line 254-265 “Sensitivity analysis demonstrated that the study of Zabicka play a role in heterogeneity. It could be influenced by the factor that the experiment performed during a VanA E. faecium outbreak, as the report of Dekeyser et al.^[24]^, and none of the patients was colonized with VanB enterococci. Several FP vanB results may be concerned with specimen type, stool swabs. Stool and rectal swabs might be the harbors where anaerobic microbes were commonly checked, which increased the risks for detecting false-positive vanB results ^[11]^.The meta regression also confirmed the specimen types might be one of possible sources of heterogeneity. Restrained by only two studies conducting both experiments on vanA and vanB detecting simultaneously or separately, a further analysis is waiting for more data.”

**③ The introduction does not do a great job of setting up the problem statement and "hows" of study.**

Response:

We agree with the reviewers regarding the matters of introduction. Hence, we have now made some improvements. Line 62-68,“It is indicated that GeneXpert vanA/vanB testing is a sensitive as well as cost-effective method as compared to the traditional culture^[5, 11]^. In addition, there some researches support that some uncertain results exist in that of GeneXpert vanA/vanB detecting van B^[10, 12]^. There are few systematic-analyses on the diagnostic accuracy of GeneXpert vanA/vanB for VRE in evidence-based medicine. Therefore, to appraise the accuracy of GeneXpert vanA/vanB in the diagnosis of VRE and discriminate the differences between GeneXpert vanA/vanB detecting vanA and vanB, we conducted a data integration analysis.”

**Results**

**① Table 1 is too large and spans multiple pages, was hard to follow. Figure Captions are insufficient.**

Response:

We regret for the problems in Table1. As a solution, we adjusted the format of Table 1, including modification of font size and presenting it on a landscape page for clarity. In addition, we checked all the submitted files, and complemented the figure captions appropriately.

**Conclusions**

**① Based on the initial comments regarding data assessment, quality and bias, it is challenging to say the conclusions are supported. All the limitations are not addressed.**
 Response:

I appreciate the opportunity to respond to the reviewer here rather than substantially changing the text. According to your advice, we acknowledge that there exist some limitations in our study. Moreover, we took some measures to reduce them to minimum.

In the first place, as having answered in questions before, we carried out a systematic literature search once more for publications. Finally, 8 articles were still maintained to analysis, evaluated the study’s eligibility based on inclusion/exclusion criteria, and assessed the methodological quality of the studies using the QUADAS-2 checklist again. In the second place, in order to endeavor minimize the existing limitations, some means were taken to address those problems. When analyzing the data, a random effects model was used to synthesis the identified studies in our analysis. Further, we supplementary meta regression and sensitivity analysis to investigate potential sources of heterogeneity. Subgroup analysis is looking forward more updating data. The results of sensitivity analysis show that one study^[1]^ (Zabicka) have an effect on heterogeneity. After omitting it, the heterogeneity decreased from 87% to 45% (<50%). We also discussed reasons of study causing heterogeneity. By means of meta regression, we investigate one of potential sources of heterogeneity was specimen type.

As line 270-279 shown: “The present study has several limitations. First, remarkable heterogeneity was observed in this study. To account for this heterogeneity, a random effects model was used to synthesis the identified studies in our analysis, which potentially increases the probability of type I error. Moreover, the results of meta regression and sensitivity analysis were attempted to explain that detected sample could partly explain the source of heterogeneity. Subgroup analysis is looking forward more updating data. Second, our study also confirmed the observation of other authors that the Cepheid Xpert vanA/vanB test has a low positive predictive value (PPV) for vanB enterococci^[26]^. Combining additional detection technologies may represent a pragmatic solution to increase VRE detection rates. Finally, we only retrieved published literature from four English databases. Only included studies written in English may have affected our findings. Despite comprehensive searches, the influence of unpublished positive results on the overall results could not be eliminated.”

Replies to Reviewer 2

Specific Comments

**① Study design is appropriate and the statistical analysis is accurate.**
Response:

Thank you for your comments. We are encouraged by the reviewers’ positive recognition of our system analysis.

**Results**

**① Figures can be improved.**
Response:

We regret for the problems in Figures. As a solution, we adjusted the format of Table 1, including modification of font size and presenting it on a landscape page for clarity. In addition, we checked all the submitted files, and complemented the figure captions appropriately.

**Conclusions**

**① Authors confirmed diagnostic accuracy of GeneXpert vanA/vanB when diagnosing Vancomycin-resistant enterococci infections. in addition, Vancomycin-resistant enterococci test shows more accuracy when diagnosing van A. As already stated by authors, the main limitations of the study are that these results are based on published data and literature where accuracy of GeneXpert vanA/vanB has been already showed and the limited sample size. Authors should better describe the implication of these results for the public health. Authors suggest major caution or additional test for detecting Van B?**
 Response:

Thank you for your suggestion. The culture method for all clinical E. faecium isolates may neither be feasible nor cost-efficient in the setting of every routine lab, which makes it impossible to make a clear decision about the need to isolate the patient. Hence, supplementary tests are needed further investigating. As already shown in our study Cepheid Xpert vanA/vanB has a high accuracy diagnosing VRE, but has a low positive predictive value (PPV) for vanB enterococci. Combining additional detection technologies may represent pragmatic solution to increase VRE detection rates, which can help healthcare workers to isolate patients more timely and effectively.

As line 63-79 shown，“It is indicated that GeneXpert vanA/vanB testing is a sensitive as well as cost-effective method^[5, 11]^. In addition, there are some researches support that some indetermination results exist in that of GeneXpert vanA/vanB detecting van B ^[10, 12]^. There are few systematic-analyses on the diagnostic accuracy of GeneXpert vanA/vanB for VRE in evidence-based medicine. Therefore, to appraise the accuracy of GeneXpert vanA/vanB in the diagnosis of VRE and distinguish the differences between GeneXpert vanA/vanB detecting vanA and vanB, we conducted data integration and analysis.”

As line 276-279 shown, “Second, our study also confirmed the observation of other authors that the GeneXpert vanA/vanB test has a low positive predictive value (PPV) for vanB enterococci[25]. Combining additional detection technologies may represent a pragmatic solution to increase VRE detection rates.”

Editorial and Data Presentation Modifications?
① **Abstract is not clearly presented. Authors should better specify that their study is not a de novo evaluation of the accuracy of GeneXpert vanA/vanB in the diagnosis of VRE, but rather than a data integration analysis of different dataset to test GeneXpert vanA/vanB accuracy and specificity in detecting Van A and Van B.**

Response：

Thank you for your suggestion. After considering your suggestion, we have improved the Abstract in order to specify what we have done in this study, shown in Line 28-33: “Experimental data were extracted from publications until May 03, 2021 related to the diagnostic accuracy of GeneXpert vanA/vanB for VRE in PubMed, Embase, Web of Science and the Cochrane Library. We used the summary receiver to operate characteristic curve (SROC curve), pooled sensitivity, pooled specificity, PLR(positive likelihood ratio), NLR (negative likelihood ratio), and DOR(diagnostic odds ratio) to evaluate the accuracy of GeneXpert vanA/vanB for VRE.”

**② Extensive english editing is required throughout the entire manuscript.**
 Response:

We regret there were problems with the English. The paper has been carefully revised by a native English speaker to improve the grammar and readability.

**Summary and General Comments**

**① The manuscript is well structured.** **Results obtained from the authors could be expected investigating the accuracy of an FDA approved diagnostic test for VRE. To this purpose I suggest authors to better describe the aim of the study. Why it is important to test Van A and Van B specificity and distinctive diagnostic accuracy if test is already approved?**

**Results obtained for Van B are enough to suggest a complementary test to be included?**

Response:

We appreciate this reminder. The sensitivity and specificity of the Cepheid Xpert vanA/vanB test as well as the positive (PPV) and negative (NPV) predictive values were the subject of several works which were included. We retrieved 4 databases again in attempt to find out more articles, but failed at presence. Therefore, as this exceeds the scope of this paper, we will bear this in mind in our future research and consider incorporating this suggestion in our research design. The culture method for all clinical E. faecium isolates may neither be feasible nor cost-efficient in the setting of every routine lab, which makes it impossible to make a clear decision about the need to isolate the patient. Hence, supplementary tests are needed further investigating. As already shown in our study Cepheid Xpert vanA/vanB has a high accuracy diagnosing VRE, but has a low positive predictive value (PPV) for vanB enterococci. Combining additional detection technologies may represent pragmatic solution to increase VRE detection rates, which can help healthcare workers to isolate patients more timely and effectively.

Replies to Reviewer 3

Specific Comments

**① Objective were clearly stated and study design was appropriate and Appropriate Statistical analysis methods were used. However the population was not clearly defined as it was given as a range from the various studies.**

Response:

We appreciate this reminder. However, in our search strategy, we have no control the population. We set ‘Humans samples were detected and analyzed’ as one of our Inclusion criteria. One of the objects is as more as impossible to acquire articles under present condition. In our study, we observed that the in our included studies is mostly tend to high-risk patients, such as those in intensive care or bone marrow transplant units or suffering from other deceases. (Studies by N. Esther Babady, 2012^[2]^; Erin S. Marner, 2011^[3]^; Dorota Zabicka, 2011^[1]^; M. Gazin & C, 2011^[4]^; B. J. Holzknecht, 2017^[5]^; M. Papadimitriou-Olivgeris, 2016^[6]^) Another population r two articles’ characters of population are no mentioned. Still, they are all human samples. What we found was consistent with the report of Prematunge C eta^[7].^ We retrieved 4 databases again in attempt to find out more articles, but failed at presence. Subgroup analysis over characters of population is looking forward more updating data.Therefore, as this exceeds the scope of this paper, we will bear this in mind in our future research and consider incorporating this suggestion in our research design.

② **Not satisfied with the final number of publications used for the analysis**

Response:

We appreciate your kindness pointing out this problem. Here are what we have done to resolve it.

We carried out a systematic literature search once more for publications in 4 databases: PubMed, Embase, Web of Science and the Cochrane Library. We make our best to retrieve articles as many as possible. According to PCIO criteria, our search stratagem was as follow: (((Enterococcus) AND (Vancomycin Resistance)) OR (Vancomycin-Resistant Enterococci)) AND (GeneXpert vanA/vanB). We also search in MeSH and Emtree for supplement of subject words and random words. Furthermore，related references had been taken into consideration. All measures were taken to avoid the omission of researches. All in all, we cannot acquire more articles under present condition. We are looking forward to more researches later to promote the conclusion.

As saying at “line 73-79”, “A systematic literature search was carried out for publications until May 03, 2021, related to the diagnostic accuracy of GeneXpert vanA/vanB for VRE. Four databases were involved: PubMed, Embase, Web of Science and the Cochrane Library. According to PCIO criteria, our search stratagem was as follow: (((Enterococcus) AND (Vancomycin Resistance)) OR (Vancomycin-Resistant Enterococci)) AND (GeneXpert vanA/vanB). Possible matches were also retrieved from the related references and the language was restricted to English.”

**Results**
**① Results were clearly and completely presented. Table 1 could be presented on a landscape page for clarity**

Response:
We regret for the problems inTable1. As a solution, we adjusted the format of Table 1, including modification of font size and presenting it on a landscape page for clarity. In addition, we checked all the submitted files, and complemented the figure captions appropriately.

**Conclusions**

**① Limitations of the study were clearly articulated and conclusions made were appropriate**
Response:

Thanks for your appreciation.

**Editorial and Data Presentation Modifications?**

**Minor Revision:**
**line 27 replace were with was
line 36 Confidence interval (0.99-0.99) not stated as a range
line 37 delete And at the start of the sentence
line 231-233-rephrase statement, too many**

Response：

We are sorry for bothering you with this kind of mistakes that we should have avoided. The paper has been carefully revised by a native English speaker to improve the grammar and readability.

**Summary and General Comments**

1. **None**
   Response:

Thank you for your comments. We are encouraged by the reviewers’ positive recognition of our system analysis.

Reference

1.	Zabicka D, Strzelecki J, Wozniak A, Strzelecki P, Sadowy E, Kuch A, et al. Efficiency of the Cepheid Xpert vanA/vanB assay for screening of colonization with vancomycin-resistant enterococci during hospital outbreak. Antonie van Leeuwenhoek, International Journal of General and Molecular Microbiology. 2012;101(3):671-5. Epub 2011/11/30. doi: 10.1007/s10482-011-9681-z.

2.	Babady NE, Gilhuley K, Cianciminio-Bordelon D, Tang YW. Performance characteristics of the Cepheid Xpert vanA assay for rapid identification of patients at high risk for carriage of vancomycin-resistant Enterococci. J Clin Microbiol. 2012;50(11):3659-63. Epub 2012/09/14. doi: 10.1128/jcm.01776-12. PubMed PMID: 22972822; PubMed Central PMCID: PMCPMC3486258.

3.	Marner ES, Wolk DM, Carr J, Hewitt C, Dominguez LL, Kovacs T, et al. Diagnostic accuracy of the Cepheid GeneXpert vanA/vanB assay ver. 1.0 to detect the vanA and vanB vancomycin resistance genes in Enterococcus from perianal specimens. Diagnostic Microbiology and Infectious Disease. 2011;69(4):382-9. Epub 2011/03/15. doi: 10.1016/j.diagmicrobio.2010.11.005.

4.	Gazin M, Lammens C, Goossens H, Malhotra-Kumar S. Evaluation of GeneOhm VanR and Xpert vanA/vanB molecular assays for the rapid detection of vancomycin-resistant enterococci. European journal of clinical microbiology & infectious diseases : official publication of the European Society of Clinical Microbiology. 2012;31(3):273-6. Epub 2011/06/15. doi: 10.1007/s10096-011-1306-y. PubMed PMID: 21667270.

5.	Holzknecht BJ, Hansen DS, Nielsen L, Kailow A, Jarløv JO. Screening for vancomycin-resistant enterococci with Xpert® vanA/vanB: diagnostic accuracy and impact on infection control decision making. New Microbes New Infect. 2017;16:54-9. Epub 2017/02/17. doi: 10.1016/j.nmni.2016.12.020. PubMed PMID: 28203378; PubMed Central PMCID: PMCPMC5295639.

6.	Papadimitriou-Olivgeris M, Filippidou S, Kolonitsiou F, Drougka E, Koutsileou K, Fligou F, et al. Pitfalls in the identification of Enterococcus species and the detection of vanA and vanB genes. Lett Appl Microbiol. 2016;63(3):189-95. Epub 2016/07/02. doi: 10.1111/lam.12610. PubMed PMID: 27367648.

7.	Prematunge C, MacDougall C, Johnstone J, Adomako K, Lam F, Robertson J, et al. VRE and VSE Bacteremia Outcomes in the Era of Effective VRE Therapy: A Systematic Review and Meta-analysis. Infect Control Hosp Epidemiol. 2016;37(1):26-35. Epub 2015/10/06. doi: 10.1017/ice.2015.228. PubMed PMID: 26434609; PubMed Central PMCID: PMCPMC4707508.
