# Supplementary material for: Evaluation of GeneXpert vanA/vanB in the early diagnosis of vancomycin-resistant enterococci infection
Source: PLoS Negl Trop Dis. 2021 Nov 8;15(11):e0009869. doi: 10.1371/journal.pntd.0009869 (PMC8575182; doi:10.1371/journal.pntd.0009869)
Supplement: Attachment — Submitted filename: Response to Reviewers.docx [file pntd.0009869.s006.docx]

Dear Dr. Alfredo G Torres,

Thanks for your letter and for reviewer’s comments concerning our manuscription entitled “"*Evaluation of GeneXpert vanA/vanB in the early diagnosis of vancomycin-resistant enterococci*" (Manuscript number: PNTD-S-20-02757).” Those comments are all valuable and helpful for revising and improving our paper. We have studied all comments carefully and have made conscientious correction. Revised portion are marked in yellow in the paper. The main corrections in the paper and the responds to the reviewer’s comments are as followed. These changes will not influence the content and framework of the paper. We appreciate for reviewers’ warm work earnestly, and hope that the correction will meet with approval.

Yours sincerely,

Xu-Guang Guo.

We would like to express our sincere thanks to the reviewers for the constructive and positive comments.

Replies to Reviewer 2

Editorial and Data Presentation Modifications?

1. **Reviewer #2: minor revision**

Response: Thank you for your suggestion. We checked all the submitted figures and tables making sure data accuracy and format consistence. In addition, we improve the format of Table 1 to enhance clarity including modification of line spacing to the same height and adding cell shading.

Summary and General Comments

1. **Reviewer #2: Authors provided most of the corrections required. Abstract has been improved but English editing still need to be performed. Manuscript should be revised by a native English speaker.**

Response: Thank you for your valuable feedback. And we regretted for the problem in English. As a solution, we ask for assistance to another professor who is a native English speaker and proficient in English manuscript editing. She polished our paper in detail to minimize typographical, grammatical, and bibliographical errors. Many modifications have been carefully revised repeatedly as highlighted in the manuscript. Hope the revised version could meet with your requirements.
